# A comparative analysis of transcriptomic, biochemical, and physiological responses to elevated ozone identifies species-specific mechanisms of resilience in legume crops

**DOI:** 10.1093/jxb/erv404

**Published:** 2015-08-31

**Authors:** Craig R. Yendrek, Robert P. Koester, Elizabeth A. Ainsworth

**Affiliations:** ^1^Institute for Genomic Biology, University of Illinois, Urbana, IL 61802, USA; ^2^Department of Plant Biology, University of Illinois, Urbana, IL 61801, USA; ^3^Global Change and Photosynthesis Research Unit, USDA ARS, Urbana, IL 61801, USA

**Keywords:** Ascorbate–glutathione cycle, air pollution, *Glycine max*, *Phaseolus vulgaris*, photosynthesis, *Pisum sativum*, RNA-Seq.

## Abstract

Current concentrations of tropospheric ozone ([O_3_]) pollution negatively impact plant metabolism, which can result in decreased crop yields. Interspecific variation in the physiological response of plants to elevated [O_3_] exists; however, the underlying cellular responses explaining species-specific differences are largely unknown. Here, a physiological screen has been performed on multiple varieties of legume species. Three varieties of garden pea (*Pisum sativum* L.) were resilient to elevated [O_3_]. Garden pea showed no change in photosynthetic capacity or leaf longevity when exposed to elevated [O_3_], in contrast to varieties of soybean (*Glycine max* (L.) Merr.) and common bean (*Phaseolus vulgaris* L.). Global transcriptomic and targeted biochemical analyses were then done to examine the mechanistic differences in legume responses to elevated [O_3_]. In all three species, there was an O_3_-mediated reduction in specific leaf weight and total non-structural carbohydrate content, as well as increased abundance of respiration-related transcripts. Differences specific to garden pea included a pronounced increase in the abundance of *GLUTATHIONE REDUCTASE* transcript, as well as greater contents of foliar glutathione, apoplastic ascorbate, and sucrose in elevated [O_3_]. These results suggest that garden pea may have had greater capacity for detoxification, which prevented net losses in CO_2_ fixation in an elevated [O_3_] environment.

## Highlight

Photosynthesis and leaf longevity were unaffected by growth in elevated [O_3_] in pea. Resilience to elevated [O_3_] was supported by transcriptional and biochemical metabolism associated with detoxification and antioxidant metabolism.

## Introduction

One of the most harmful air pollutants impacting plant growth today is tropospheric ozone (O_3_; Krupa *et al.*, 2001). Over the past 100 years, ground-level O_3_ concentrations ([O_3_]) have increased four-fold (Seinfeld and Pandis, 2012) and may continue to increase in the absence of precursor gas emission reductions (Jacobson and Streets, 2009). Upon entry of O_3_ into leaves, formation of reactive oxygen species (ROS) occurs in the apoplast, triggering enhanced antioxidant metabolism (Sharma and Davis, 1997; Mittler, 2002), which is hypothesized to be driven by an increase in mitochondrial respiration (Amthor 1988; Ainsworth *et al.*, 2012). Exposure to [O_3_] that exceed the capabilities of the cellular detoxification system leads to damage of membranes and proteins, including components of the photosynthetic machinery, resulting in decreased CO_2_ assimilation (Ashmore, 2005; Fiscus *et al.*, 2005). Worldwide, the metabolic changes induced by current [O_3_] are responsible for reductions in crop productivity estimated to cost US$11–26 billion annually (Van Dingenen *et al.*, 2009; Avnery *et al.*, 2011).

Characterizing the underlying molecular changes that determine the metabolic responses to elevated [O_3_] has been an active area of research for decades, especially related to ROS scavenging (Sandermann *et al.* 1998; Schraudner *et al.*, 1998; Rao *et al.*, 2000; Wohlegemuth *et al.*, 2002). The abundance and redox states of the antioxidants ascorbate and glutathione are important for detoxification of O_3_-induced ROS (Foyer and Noctor, 2005), and act to prevent cellular damage and maintain normal metabolic activity. In addition, proper regulation of the suite of enzymes comprising the ascorbate–glutathione cycle is necessary for recycling oxidized ascorbate and glutathione back to the reduced form (Noctor and Foyer, 1998). Global gene expression and proteomics studies have identified antioxidant transcripts and proteins with increased content in response to elevated [O_3_] (Agrawal *et al.*, 2002; Baier *et al.*, 2005). Transgenic approaches have also been used to alter the abundance and/or redox state of the ascorbate and/or glutathione pools in order to better understand the mechanisms of cellular O_3_ response. These studies have highlighted the complexity of O_3_-induced oxidative signalling, which depends on the coordinated expression of specific ascorbate–glutathione cycle isoenzymes in the correct subcellular compartments to maintain redox homeostasis (Noctor and Foyer, 1998; Foyer and Noctor, 2005). For example, tobacco plants overexpressing a manganese *SUPEROXIDE DISMUTASE* (*SOD*) gene in the chloroplast showed reduced injury in response to elevated [O_3_] (Van Camp *et al.*, 1994), while no protection was afforded by overexpression of a chloroplastic copper or zinc *SOD* (Pitcher *et al.*, 1991). Complicating the comparison between these two studies are different O_3_ exposures (concentration and duration) used to screen the transgenic plants.

Soybean (*Glycine max* (L.) Merr.) is the world’s most widely grown leguminous crop, and is classified as being highly responsive to [O_3_] (Mills *et al.*, 2007). Soybean seed yield is significantly reduced by [O_3_] exceeding 40 nL L^−1^ (Heagle, 1989), and decreased photosynthesis is thought to be one of the major determinants of yield loss (Betzelberger *et al.*, 2012). In addition, antioxidant metabolism and mitochondrial respiration are increased in soybean exposed to elevated [O_3_] (Gillespie *et al.*, 2012). While some studies have reported intraspecific variation for the response of soybean to elevated [O_3_] (Lee *et al.*, 1984; Burkey and Carter, 2009; Betzelberger *et al.*, 2010), others have found no difference between varieties and that a single response function can accurately describe soybean O_3_ responses (Mills *et al.*, 2007; Betzelberger *et al.*, 2012). If most soybean varieties are negatively impacted by elevated [O_3_], then a wider examination of species that fail to display typical negative responses to elevated [O_3_] might reveal novel cellular strategies that could be used in soybean improvement efforts.

The goal of this study was to characterize cellular changes in leaves of diverse legume species demonstrating a broad range of O_3_-induced physiological and growth responses. Previous physiological comparisons have revealed that photosynthesis is not universally affected by growth at elevated [O_3_] in some legumes. For example, several photosynthetic parameters in garden pea (*Pisum sativum* L.) were not significantly reduced by growth in chronic [O_3_] of 80 nL L^−1^ (Farage and Long, 1999). On the other hand, common bean (*Phaseolus vulgaris* L.) varieties have been developed for use as O_3_ bioindicators, and moderate increases in [O_3_] (to 50 nL L^−1^) can decrease yields by up to 50% in some varieties (Burkey *et al.*, 2005). Using a comparative approach, several varieties of soybean, garden pea, and common bean were first screend to identify genotypes exhibiting a broad range of photosynthetic responses to elevated [O_3_]. RNA sequencing (RNA-Seq) was then used to compare the global transcriptomic response with changes in antioxidant metabolite content of the selected legumes, and cellular responses associated with the O_3_-induced photosynthetic response were identified.

## Materials and methods

### Plant growth conditions

Seeds of commercially available garden varieties of garden pea (*Pisum sativum* L.), soybean (*Glycine max* (L.) Merr.), and common bean (*Phaseolus vulgaris* L.) were planted in 6L pots (21.6cm tall; 22.9cm diameter) containing sterile soilless media (LC1 Sunshine mix; Sun Gro Horticulture Distribution Inc., Bellevue, WA, USA). All varieties were short-season varieties, with maturity dates ranging from 56 to 62 days after planting for pea, 50 to 53 days for common bean, and 80 to 82 days for soybean. Three pots of each species were placed in each of six growth chambers (Environmental Growth Chamber, Chagrin Falls, OH, USA) set to maintain constant conditions for light (900 µmol m^−2^ s^−1^; 16h d^−1^), temperature (24°C day; 21°C night), and relative humidity (60%). Plants were watered as needed and fertilized once per week with water-soluble plant food (Miracle-Gro, Scotts Company LLC, Marysville, OH, USA). Three chambers were fumigated with an average [O_3_] of 151.2 nL L^−1^ ± 0.72 nL L^−1^ for 8h d^−1^, starting 4h after the start of the light period, throughout the duration of the experiment. O_3_ was generated and controlled as described in Yendrek *et al.* (2013). The other three chambers were maintained at ambient levels of O_3_, with an average [O_3_] of 12.5 nL L^−1^ ± 0.96 nL L^−1^. To determine leaf longevity, the date (approximately 9 d after planting; DAP) at which the third leaf of pea and the first trifoliate of soybean and common bean had elongated >0.5cm was subtracted from the date that leaf abscission was observed. At the conclusion of the experiment (45 DAP), plants were destructively harvested and leaf area was measured with a LI-3100C area meter (LI-COR, Lincoln, NE, USA). Leaves were then dried at 60°C for 48h and weighed.

### Gas exchange measurements

**Fig. 1.**
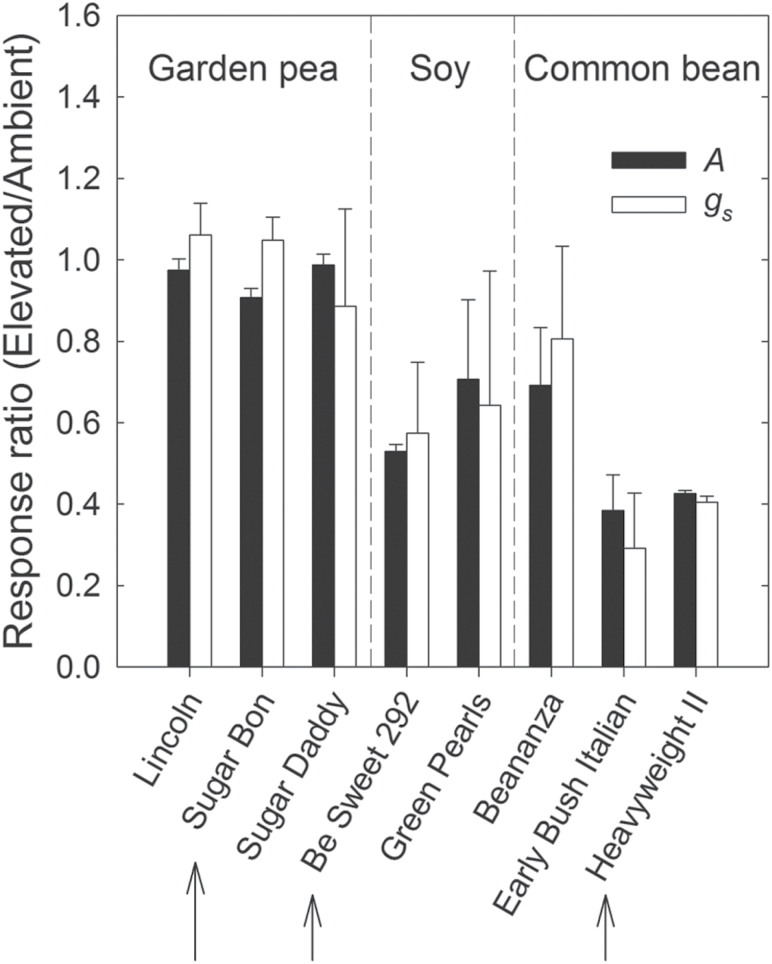
The response (± SD; n = 3) of net assimilation (*A*) and stomatal conductance (*g*_*s*_) to elevated [O_3_] in varieties of garden pea, soybean, and common bean. *In situ* gas exchange measurements were performed at midday on the youngest, fully expanded leaf after growth in elevated [O_3_] (150 ppb; 8h d^−1^) for one month. Arrows indicate varieties used for additional comparative studies.

For the photosynthesis screen, *in situ* net assimilation (*A*) and stomatal conductance (*g*_*s*_) were measured on the youngest fully expanded leaf (pea, seventh leaf; soybean, fourth trifoliate; common bean, third trifoliate) of all plants in every chamber 30 DAP at midday, approximately 8h after the lights were turned on in the chambers (4h after the start of O_3_ fumigation). For this, an infrared gas analyzer (LI-6400, LI-COR) was set to match the ambient growth conditions, including temperature (block temperature, 24°C), relative humidity (60%), light (900 µmol m^−2^ s^−1^), and [CO_2_] (400 µL L^−1^). In another experiment with varieties selected from the screen (Fig. 1), one plant per species in each chamber was used to estimate maximum Rubisco carboxylation capacity (*V*_*c,max*_) and maximum potential linear electron flux (*J*_*max*_) 22 DAP. *A* was measured across a range of internal [CO_2_] (*C*_i_) (from 50 µL L^−1^ to 1500 µL L^−1^) at saturating light (1300 µmol m^−2^ s^−1^) and ambient growth conditions for temperature (24°C) and relative humidity (60–65%). The values for *A* and *C*_i_ were fit to a C_3_ model of photosynthesis (Farquhar *et al.*, 1980) following the methods of Long and Bernacchi (2003). All leaves sampled had emerged 11–13 DAP and were the youngest fully expanded leaves (pea, fifth leaf; soybean, third trifoliate; common bean, second trifoliate).

### Quantification of primary metabolites and ROS scavenging molecules

At 34 DAP, one leaflet was collected from the same cohort of leaves used to measure *in situ* gas exchange, frozen in liquid N, and ground to a fine powder. Tissue from three individual plants per chamber was pooled for each species and approximately 50mg was used to quantify total non-structural carbohydrate (TNC) content, including glucose, fructose, sucrose, and starch as described in Yendrek *et al.* (2013). Another 50mg aliquot of tissue was used to determine total foliar phenolic content. Briefly, phenolic compounds were extracted in 95% methanol at room temperature for 48h. The leaf extract was then incubated with 10% (v/v) Folin–Ciocalteu solution and 700mM Na_2_CO_3_ at room temperature for 2h. To calculate total phenolic content, the absorbance of each sample was measured at 765nm and values were compared to a curve of gallic acid standards (Ainsworth and Gillespie, 2007). To quantify glutathione content, approximately 10mg of ground leaf tissue was mixed with 1× phosphate buffered saline with 2mM EDTA (pH 8.0). Total and oxidized glutathione content was assayed using a GSH/GSSG-Glo Assay kit following the manufacturer’s protocol (Promega Corporation, Madison, WI, USA). Quantification of total and reduced ascorbate was determined following the methods of Gillespie and Ainsworth (2007) using approximately 30mg of ground leaf tissue or 50 µL of apoplastic fluid. Apoplastic fluid was collected from leaves cut in half along the middle vein that was vacuum-infiltrated with 2% metaphosphoric acid with 2mM EDTA, placed in a conical tube, and centrifuged at 2700 *g* for 10min at 4°C (Eller and Sparks, 2006). Apoplastic fluid was immediately frozen in liquid N and stored at −80°C until assayed.

### Statistical analysis of whole plant, physiological, and biochemical results

An initial analysis using PROC UNIVARIATE (SAS, Version 9.2, Cary, NC, USA) was performed to establish that data were normally distributed. A two-way ANOVA using PROC GLM (SAS) was then done to assess the impact of elevated [O_3_] on the three legume species. Pairwise comparisons of the least squares means were performed to identify significant differences.

### RNA isolation and sequencing

Total RNA was purified from the same tissue used for biochemical analyses following the procedure of Bilgin *et al.* (2009). High levels of polysaccharide co-precipitated with the RNA in the common bean and pea samples, so Plant RNA Reagent (Life Technologies, Grand Island, NY, USA) was then used according to the manufacturer’s instructions. All RNA samples were DNase-treated with a TURBO DNA-free kit (Life Technologies) and separated on an agarose gel to verify quality (Supplementary Fig. S1). Each RNA sample was individually barcoded during cDNA library preparation using the TruSeq RNA Sample Prep kit (Illumina, San Diego, CA, USA) and pooled (four samples per lane) for single-end sequencing on a HiSeq2000 (Illumina) for 100 cycles. Sequencing data were submitted to NCBI [GenBank: SRP009826].

### Determination of differential gene expression

Before aligning to the reference transcriptomes, duplicate reads (identical sequences as well as those that mapped to more than one transcriptome location) were removed and the remaining reads were filtered with the FASTQ Quality Filter (FASTX-Toolkit; Gordon and Hannon, unpublished) to keep reads that had a minimum quality score of 20 across at least 90% of the read length. Reads were aligned using the --b2-sensitive option for Tophat2 / Bowtie2 (Langmead *et al.*, 2009; Langmead and Salzberg, 2012). The reference transcriptome sequence file for pea (*Pisum sativum* unigene v2) was obtained from the Cool Season Food Legume Genome Database (https://www.coolseasonfoodlegume.org). For soybean (Gmax_189_transcript.fa; Schmutz *et al.*, 2010) and common bean (Pvulgaris_218_transcript.fa; Schmutz *et al.*, 2014), the reference transcriptome files were obtained from Phytozome (http://www.phytozome.net/). After sorting and converting the BAM alignment output file with SAMtools (Li *et al.*, 2009), the Python package HTSeq (www-huber.embl.de/users/anders/HTSeq/doc/overview.html) was used to generate read counts for each gene using the intersection-nonempty mode. These counts were then analyzed by the R package edgeR, using the TMM normalization, to generate a list of differentially expressed genes (Robinson *et al.*, 2010). Summary statistics for the bioinformatics analysis are presented in Supplementary Table S1.

## Results

### Species-specific growth and physiological responses to elevated [O_3_]

A physiological screen of several garden varieties of pea, soybean, and common bean showed no major reductions in *A* and *g*_*s*_ in any of the garden pea varieties (Fig. 1). In contrast, each of the soybean and common bean varieties had lower *A* and *g*_*s*_ (Fig. 1). ‘Early Bush Italian’ and ‘Heavyweight II’, both varieties of common bean, were most responsive to growth in elevated [O_3_], with reductions in *A* and *g*_s_ of ~60% (Fig. 1).

**Fig. 2.**
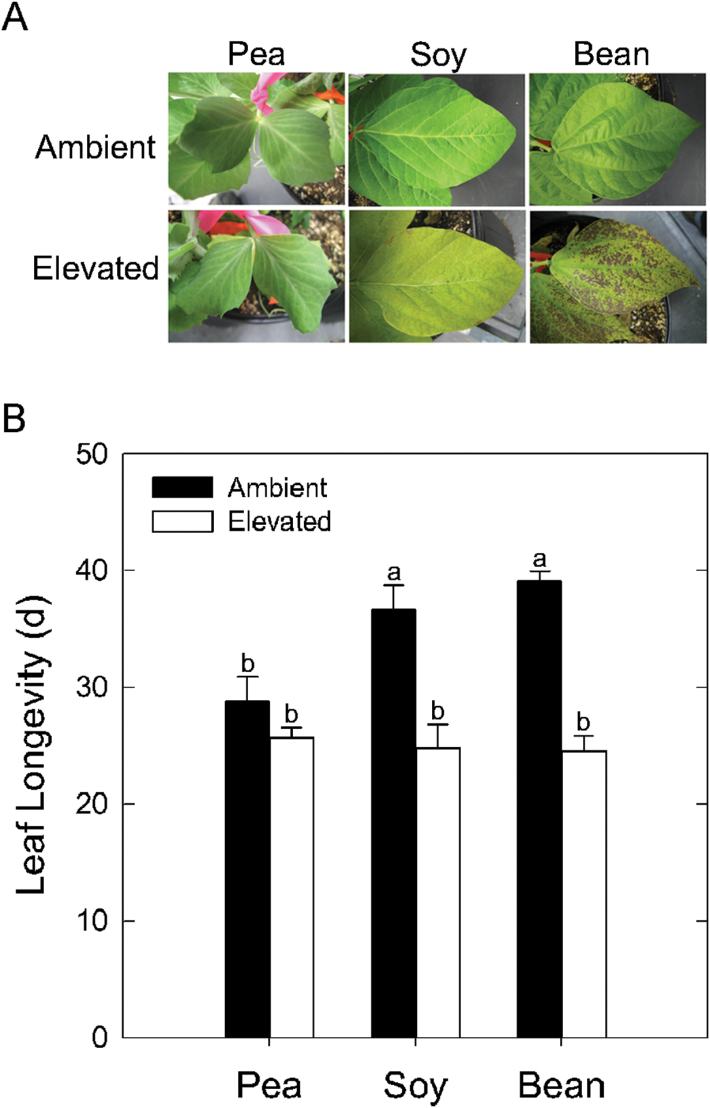
Interspecific comparison of leaf and whole plant parameters, including (A) visual observations following 6 weeks of growth in elevated [O_3_] (150 ppb; 8h d^−1^) and (B) leaf longevity, determined as the number of days between leaf primordia emergence (<0.5cm long) and leaf abscission. The mean (± SD; n = 3) is presented with letters representing significant differences (*P* < 0.05).

**Fig. 3.**
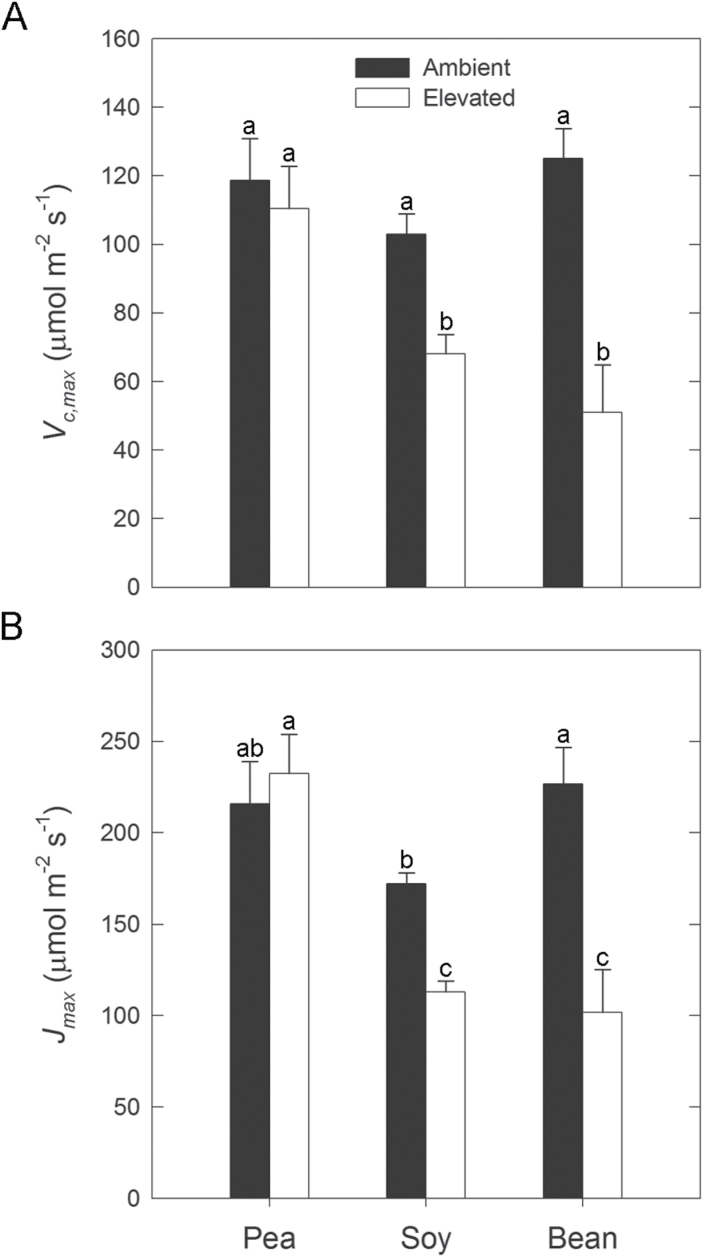
Leaf-level gas exchange parameters, including estimates of (A) maximum carboxylase activity, *V*_*c,max*_, and (B) photosynthetic electron transport, *J*_*max*_. The mean (± SD; n = 3) is presented with letters representing significant differences (*P* < 0.05).

In order to more thoroughly compare leaf responses to elevated [O_3_], one representative variety of each legume species, including ‘Sugar Bon’ (garden pea), ‘Be Sweet 292’ (soybean), and ‘Heavyweight II’ (common bean), was investigated further. Garden pea displayed no visual signs of O_3_ damage in contrast to soybean and common bean, which both had signs of chlorosis (Fig. 2A). More extensive O_3_ damage was observed in common bean, including leaf bronzing and necrosis. No significant change in leaf longevity was detected in garden pea grown in elevated [O_3_], while a 12–15 day decrease in soybean and common bean was seen (Fig. 2B). Soybean and common bean also had O_3_-induced decreases in photosynthetic capacity, with reductions in *V*_c,max_ and *J*_*max*_ exceeding 60% in common bean (Fig. 3). In garden pea, however, estimates of *V*_*c,max*_ and *J*_*max*_ were not decreased by elevated [O_3_] (Fig. 3).

### Global gene expression changes in legumes exposed to elevated [O_3_]

A transcriptome-wide comparison of differentially expressed genes revealed that each legume species had a distinct response to elevated [O_3_]. When considering differentially expressed genes with a log_2_ fold change >±2.0, garden pea had a greater number of genes (63% of total differentially expressed genes) showing an increase in transcript abundance. In contrast, soybean had a greater number of genes (57% of total differentially expressed genes) with decreased transcript abundance while common bean had a similar number of increased and decreased genes (Supplementary Fig. S2). There was also a difference in the genes expressed in elevated [O_3_] that had no detectable transcripts in ambient O_3_ conditions. For each species, these genes turned ‘on’ by elevated [O_3_] included peroxidases and receptor-like kinases (Supplementary Table S2). Additionally, the expression of numerous stress-related genes and transcription factors was induced by elevated [O_3_] in garden pea (Supplementary Table S2).

The distribution of differentially expressed genes across major functional categories revealed species differences in the number of genes related to protein processes (Supplementary Fig. S3A). Because the total number of genes comprising a functional category was different for each species, the percentage of genes that were differentially expressed per functional category is reported. Compared to soybean and common bean, garden pea had a greater percentage of differentially expressed genes involved with protein synthesis, post-translational modification, and protein degradation (Supplementary Fig. S3B). The majority of these genes in garden pea increased in abundance, suggesting enhanced functionality for these processes.

### Elevated [O_3_] alters antioxidant transcripts and metabolites

**Fig. 4.**
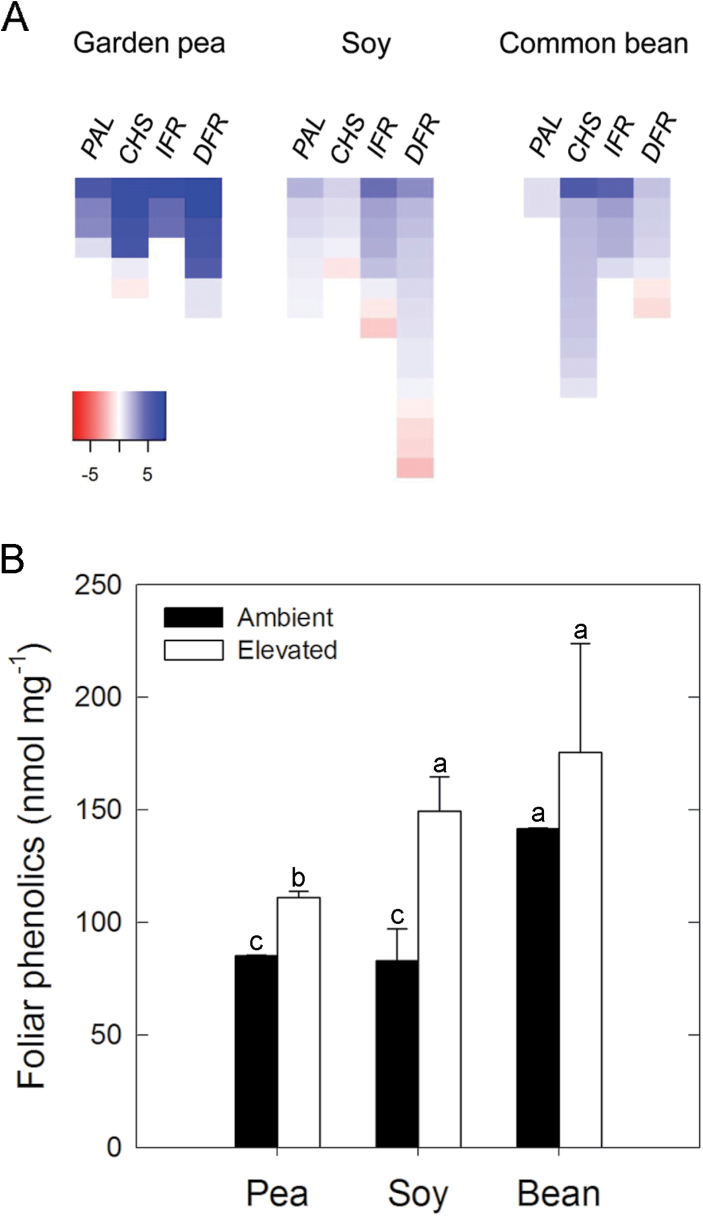
Species comparison of phenolic-related transcript and content changes in response to elevated [O_3_]. (A) Heat map of transcript abundance changes for *PHENYLANLANINE AMMONIA LYASE* (*PAL*), *CHALCONE SYNTHASE* (*CHS*), *ISOFLAVONE REDUCTASE* (*IFR*), and *DIHYDROFLAVONOL 4-REDUCTASE* (*DFR*) in leaf tissue collected from the youngest fully expanded leaf at midday after plants were grown in elevated [O_3_] (150 ppb; 8h d^−1^) for one month. Each coloured block represents the log_2_ fold change of a separate homologue that was determined to be differentially expressed. (B) Midday total foliar phenolic content of the youngest fully expanded leaf after plants were grown in elevated [O_3_] for one month. Absolute values represent gallic acid equivalents based on a comparison to a standard curve and were normalized to tissue dry weight. The mean (± SD; n = 3) is presented with letters representing significant differences (*P* < 0.05).

Markers at key branch points in phenylpropanoid metabolism, including *PHENYLANLANINE AMMONIA LYASE*, *CHALCONE SYNTHASE*, *ISOFLAVONE REDUCTASE*, and *DIHYDROFLAVONOL 4-REDUCTASE*, showed increased transcript abundance in all three legume species, but the log_2_ fold changes were greatest in garden pea (Fig. 4A). These transcriptional changes were indicative of a general up-regulation of the entire phenylpropanoid pathway and consistent with the trend of increased total foliar phenolic content (Fig. 4B). Only common bean failed to show a statistically significant increase in phenolic content, which may have been due to the small increase in *PAL* transcript abundance (Fig. 4A) at the entry point into the pathway and/or the large variation in phenolic content measured at elevated [O_3_] (Fig. 4B).

The abundance of transcripts encoding proteins involved in ascorbate–glutathione cycling was also investigated. Multiple genes encoding *ASCORBATE OXIDASE (AO*), which is localized to the cell wall, were identified in each species. At least one transcript in each species increased in plants grown at elevated [O_3_] (Table 1), with pea showing a 5.45-fold increase in expression (Table 1). However, all three species also had *AO* transcripts that either decreased or did not change in response to elevated [O_3_] (Table 1). Transcripts encoding two genes, *GLUTATHIONE PEROXIDASE 6 (GPX6*) and *SOD2* (Fe, chloroplast), were significantly increased in soybean and common bean in response to elevated [O_3_], but not in garden pea (Table 1). No evidence of transcript abundance changes [log_2_ fold change > 1.0; reads per kilobase of transcript per million reads mapped (RPKM) > 5.0] for several ascorbate–glutathione cycle genes, including *ASCORBATE PEROXIDASE*, *DEHYDROASCORBATE REDUCTASE*, *MONODEHYDROASCORBATE REDU CTASE,* and *CATAL ASE*, were observed in any species (Supplementary Table S3). The only ascorbate–glutathione cycle gene that had increased transcript abundance exclusively in garden pea was a cytoplasm-localized *GLUTATHIONE-DISULFIDE REDUCTASE* (*GR*; Table 1).

**Table 1.** Transcript abundance changes of selected ascorbate–glutathione cycle genes demonstrating conserved and distinct species responses to elevated [O_3_]. Transcript sequences of pea and common bean (Bean) were compared to soybean (Soy) using BLAST, and the most closely related transcripts based on primary sequence are grouped together. Abundance values are presented as reads per kilobase of transcript per million mapped reads (RPKM). Transcripts in bold are significantly different (false discovery rate adjusted *P* ≤ 0.05). Supplementary Table S3 shows the full list of ascorbate–glutathione cycle genes. ns, not significant.

	Species	Transcript ID	Log_2_ fold change	Ambient RPKM	Elevated RPKM
*ASCORBATE OXIDASE* [cell wall]	**Soy**	**Glyma05g33470.1**	**2.93**	**3.37**	**25.93**
	**Pea**	**ID296830_p.sativum_wa1_contig18841**	**5.45**	**1.99**	**84.39**
	Soy	Glyma13g03650.1	-0.31 (ns)	18.32	15.14
	**Soy**	**Glyma20g12150.1**	**-0.99**	**21.01**	**10.70**
	**Bean**	**Phvul.006G011600.1**	**1.11**	**22.53**	**46.41**
	**Bean**	**Phvul.006G011700.1**	**-1.92**	**19.68**	**4.94**
	Pea	ID_Pisum_sativum_v2_Contig4699	-0.12 (ns)	145.03	129.09
*GLUTATHIONE REDUCTASE* [cytoplasm]	Soy	Glyma16g27210.1	-0.11 (ns)	1.08	1.00
	Bean	Phvul.004G083700.1	0.21 (ns)	65.64	72.45
	**Pea**	**ID_Pisum_sativum_v2_Contig8768**	**1.23**	**97.60**	**224.23**
*GLUTATHIONE PEROXIDASE 6*	**Soy**	**Glyma01g42840.1**	**0.84**	**71.66**	**131.13**
	Soy	Glyma05g37900.1	0.35 (ns)	13.37	17.33
	Pea	ID_Pisum_sativum_v2_Contig1327	0.19 (ns)	7.15	7.95
	Soy	Glyma08g01700.1	0.03 (ns)	102.18	106.75
	**Bean**	**Phvul.002G288700.1**	**1.34**	**173.55**	**417.89**
	Soy	Glyma11g02630.1	0.37 (ns)	47.88	63.38
*SUPEROXIDE DISMUTASE 2* [Fe; chloroplast]	Soy	Glyma10g33710.1	0.26 (ns)	52.80	64.44
	**Bean**	**Phvul.007G135400.1**	**1.16**	**35.61**	**75.98**
	Soy	Glyma10g33710.2	0.47 (ns)	7.33	10.16
	**Soy**	**Glyma20g33880.2**	**0.53**	**346.98**	**512.39**
	**Bean**	**Phvul.007G135400.2**	**0.74**	**2.32**	**3.72**
	Soy	Glyma10g33710.1	0.26 (ns)	52.80	64.44

Because GR requires NADPH as a co-factor to regenerate reduced glutathione (Halliwell and Foyer, 1978), changes in abundance of transcripts involved in the oxidative branch of the pentose phosphate pathway were also examined. Garden pea transcript ID262852_p.sativum_wa1_contig18926, encoding a cytosolic *GLUCOSE-6-PHOSPHATE DEHYDROGENASE* (*G6PDH*) was significantly increased (log2FC of 1.91; ambient RPKM, 23.28; elevated RPKM, 85.77), whereas no *G6PDH* transcripts were differentially expressed in soybean or common bean. Increased transcript abundance of *γ-GLUTAMYL TRANSPEPTIDASE* (*GGT*), a gene that mediates glutathione degradation (Storozhenko *et al.*, 2002), was also observed in garden pea (Supplementary Table S4). Genes involved with glutathione biosynthesis were not differentially expressed in any species (Supplementary Table S4).

In garden pea, the content of total foliar glutathione was 2.9- to 4.2-fold higher in ambient [O_3_] and 1.7- to 6.1-fold higher in elevated [O_3_] compared to soybean and common bean (Table 2). Only soybean increased glutathione content in response to elevated [O_3_]. Soybean was also the only species that showed a global decrease in the extent to which proteins were glutathionylated (Supplementary Table S5), a post-translational modification that protects proteins from oxidation (Zechmann, 2014). The redox state of the glutathione pool varied among species, with garden pea having significantly less reduced glutathione as a proportion of total glutathione in elevated [O_3_] compared to soybean and common bean (Table 2). Of the three species, common bean had the greatest percentage of reduced glutathione in elevated [O_3_], as well as the lowest total glutathione content (Table 2).

**Table 2.** Midday foliar and apoplastic antioxidant content of the youngest fully expanded leaf after plants were grown in elevated [O_3_] (150 ppb; 8h d^−1^) for one month The mean (± SD; n = 3) is presented with letters representing significant differences (*P* < 0.05). FW, fresh weight; NA, data not available

	Garden pea		Soybean		Common bean	
***Foliar***	Ambient	Elevated	Ambient	Elevated	Ambient	Elevated
Total glutathione [nmol (g FW)^−1^]	947±242.5^a^	905±131.1^a^	295±8.9^c^	531±21.0^b^	193±22.6^c^	96±12.6^c^
Reduced glutathione (%)	78±9.5^bc^	69±6.1^c^	76±4.6^bc^	86±8.3^ab^	99±1.1^a^	91±8.6^a^
***Foliar***						
Total ascorbate [µmol (g FW)^−1^]	7.1±0.25^b^	6.5±1.75^b^	6.3±0.97^b^	10.0±0.90^a^	7.2±1.12^b^	7.0±1.60^b^
Reduced ascorbate (%)	100±0.0^a^	100±0.0^a^	100±0.0^a^	92±8.5^ab^	84±8.7^b^	60±1.0^c^
***Apoplastic***						
Total ascorbate [µmol (g FW)^−1^]	1.6±0.32^a^	1.8±0.58^a^	NA	NA	1.8±0.37^a^	0.2±0.15^b^
Reduced ascorbate (%)	100±0.0^a^	100±0.0^a^	NA	NA	95±6.3^a^	70±11.7^b^

Soybean also increased foliar ascorbate content in elevated [O_3_], in contrast to pea and common bean (Table 2). In addition, no change in total apoplastic ascorbate content was seen in garden pea. However, common bean showed a pronounced decrease in apoplastic ascorbate content in response to elevated [O_3_] (Table 2). Common bean also showed an O_3_-mediated decrease in the proportion of reduced ascorbate in both foliar and apoplastic pools, whereas the pools of ascorbate in garden pea and soybean were maintained nearly completely in the reduced state (Table 2).

### Elevated [O_3_] alters transcription of respiratory genes and reduces TNC content

**Fig. 5.**
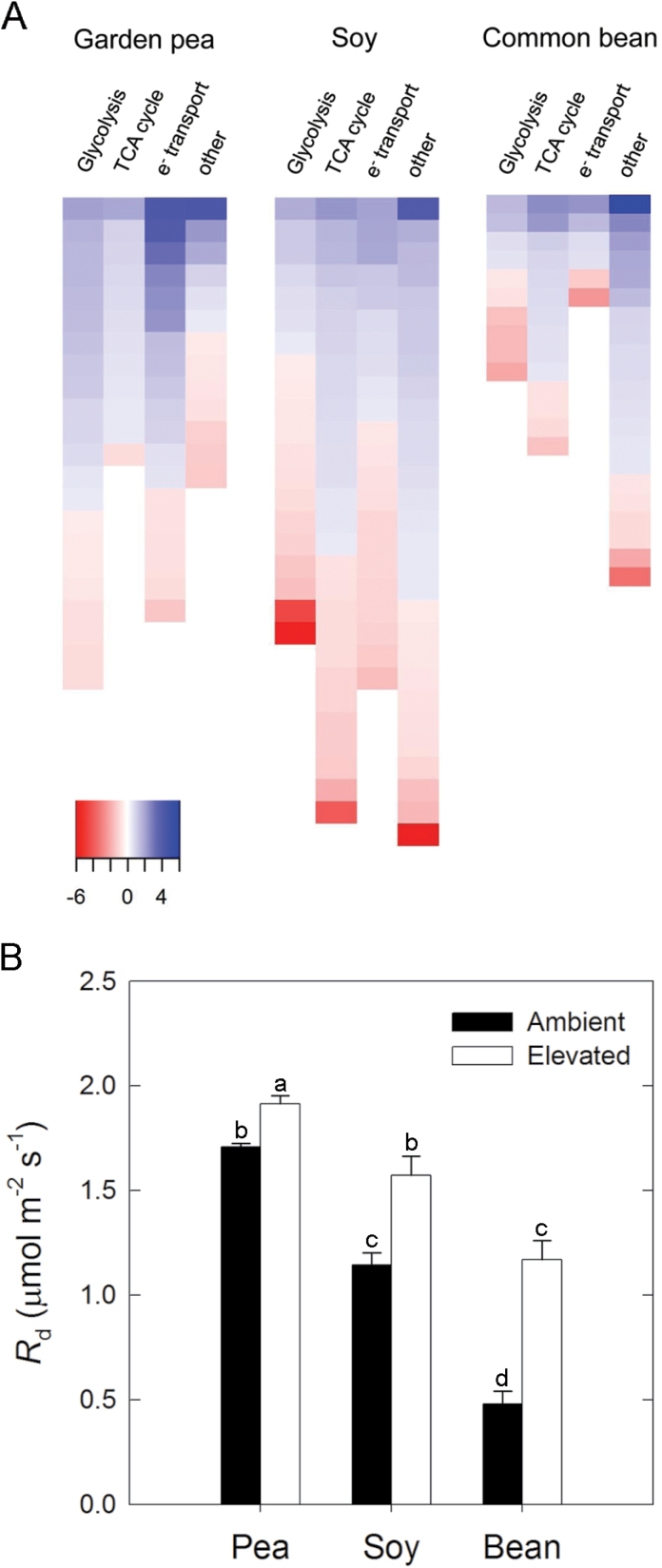
Species comparison of respiration-related transcript and metabolic changes in response to elevated [O_3_]. (A) Heat map of transcript abundance changes for genes comprising the major respiratory pathways in leaf tissue collected from the youngest fully expanded leaf at midday after plants were grown in elevated [O_3_] (150 ppb; 8h d^−1^) for one month. Each coloured block represents the log_2_ fold change of a separate homologue that was determined to be differentially expressed. (B) The rate of mitochondrial respiration in the light (*R*_d_) estimated from *A*/*C*_*i*_ curves. The mean (± SD; n = 3) is presented with letters representing significant differences (*P* < 0.05).

In each species, many respiration-related genes showed increased transcript abundance in response to elevated [O_3_] (Fig. 5A). Estimates of mitochondrial respiration in the light (*R*_d_) also indicated a stimulatory effect due to elevated [O_3_] (Fig. 5B). A closer examination revealed that, compared to soybean and common bean, garden pea had a marked increase in mitochondrial electron transport-related transcripts (Fig. 5A), including several NADH-dehydrogenase genes (Supplementary Table S6). In contrast, soybean and common bean had larger O_3_-induced decreases in several glycolysis- and tricarboxylic acid (TCA) cycle-related transcripts (Fig. 5A; Supplementary Table S6). In all three species, a large increase in the abundance of transcripts encoding *ALDEHYDE DEHYDROGENASE (ALDH*) was observed (Fig. 5A, other; Supplementary Table S6). Only in common bean were several genes in the glyoxylate cycle, including *ISOCITRATE LYASE* and *MALATE SYNTHASE*, shown to have increased transcript abundance in response to elevated [O_3_] (Fig. 5A, other; Supplementary Table S6).

Midday TNC content was significantly decreased in all three species by elevated [O_3_], as was specific leaf weight (SLW) (Table 3). Foliar starch content, the most abundant TNC constituent, showed a response similar to TNC and SLW (Table 3). Foliar sucrose content did not change in response to elevated [O_3_] in any species and was most abundant in garden pea (Table 3). Foliar glucose content was low in all three species but showed a significant O_3_-mediated increase in common bean (Table 3).

**Table 3.** Midday SLW and foliar content of TNC and constituents of the youngest fully expanded leaf after plants were grown in elevated [O_3_] (150 ppb; 8h d^−1^) for one month Absolute values represent glucose equivalents based on a comparison to a standard curve and were normalized to the amount of tissue per unit leaf area. The mean (± SD; n = 3) is presented with letters representing significant differences (*P* < 0.05).

	Garden pea		Soy		Common bean	
	Ambient	Elevated	Ambient	Elevated	Ambient	Elevated
SLW (g m^−2^)	81.0±8.90^a^	54.8±4.55^b^	64.7±1.40^b^	38.3±1.78^c^	88.0±7.39^a^	51.6±4.14^bc^
TNC (µmol cm^−2^)	53.5±13.23^a^	32.3±10.10^b^	60.1±10.32^a^	19.9±10.63^bcd^	25.6±0.95^bc^	11.1±6.17^d^
Glucose (µmol cm^−2^)	1.5±0.83^ab^	1.7±0.79^a^	0.9±0.34^b^	1.2±0.32^ab^	0.1±0.01^c^	0.7±0.22^b^
Sucrose (µmol cm^−2^)	8.4±1.25^a^	9.2±4.33^a^	2.1±0.08^b^	2.1±0.39^b^	0.8±0.01^b^	3.2±2.29^b^
Starch (µmol cm^−2^)	42.8±10.02^a^	21.3±5.41^b^	57.1±10.54^a^	16.8±10.29^bc^	24.7±0.94^b^	7.0±3.82^c^

## Discussion

Across the three legume species, there were distinct transcriptional, biochemical, and physiological responses to elevated [O_3_]. The reductions in photosynthetic parameters (Figs 1 and 3) and leaf longevity (Fig. 2) observed in soybean and common bean are typical of herbaceous annuals (Ainsworth *et al.*, 2012) and similar to previous reports in which the response to elevated [O_3_] was greater in common bean than soybean (Feng and Kobayashi, 2009). Because garden pea did not show these symptoms of O_3_ damage, we hypothesized that it employed a more effective strategy to detoxify O_3_-induced ROS. While a previous study using a single variety of garden pea reported no O_3_-induced reductions in photosynthetic capacity (Farage and Long, 1999), here it was demonstrated that this may be a more general response for the species because none of the garden pea varieties investigated exhibited a negative response to O_3_ (Fig. 1). The differences in physiological and visual symptoms of O_3_ exposure between the legumes were unlikely related to flux of the gas into the leaf. *g*_s_ was not significantly different among the three legume species in ambient [O_3_] (data not shown), and, in contrast to soybean and common bean, there was no change in *g*_*s*_ in response to O_3_ exposure in garden pea (Fig. 1). Thus, a reduction in O_3_ flux into leaves cannot explain the lack of responsiveness in garden pea; rather, the inherent capacity to activate a cellular response to elevated [O_3_] is a more likely mechanism.

Many genes in each species were turned on by elevated [O_3_], including peroxidases and receptor-like kinases (Supplementary Table S2), both of which are known to be transcriptionally regulated by abiotic stress (Noctor and Foyer, 1998, Osakabe *et al.*, 2013). The induction of MYB and WRKY family genes, which mediate transcriptional reprogramming in response to abiotic stresses including elevated [O_3_] (Tosti *et al.*, 2006; Rizzo *et al.*, 2007; Chen *et al.*, 2012; Iyer *et al.*, 2013; Li *et al.*, 2014), suggests that garden pea may have activated signalling networks involved with stress perception to a greater extent than soybean and common bean. In addition, a broad functional analysis revealed that garden pea had a greater stimulation of genes involved with protein processes (Supplementary Fig. S3). While it is known that growth in elevated [O_3_] can damage proteins and trigger proteolysis and protein biosynthesis (Pell *et al.*, 1997), the implication of the species comparison is that garden pea may have removed and replaced damaged proteins more efficiently. Considering that each species had a large number of significantly expressed genes involved with post-translational modifications, the extent of glutathionylation, which can protect proteins from irreversible oxidative damage and be stimulated by ROS (Foyer and Noctor, 2005; Rouhier *et al.*, 2008; Zechmann, 2014) was also examined. While no specific proteins were investigated, the O_3_-mediated decrease in total glutathionylated proteins in soybean (Supplementary Table S5) suggests limited protective benefit and could possibly be a contributing factor in the responsiveness of soybean to elevated [O_3_].

In all three species, the abundance of transcripts involved with the biosynthesis of phenylpropanoid compounds increased in response to elevated [O_3_] (Fig. 4A). Products of the phenylpropanoid pathway are involved with photoprotection in response to stress (Dixon and Paiva, 1995) as well as direct ROS scavenging (Rice-Evans *et al.*, 1997; Grace and Logan, 2000). Generally, the expression and abundance of phenolic metabolites are induced by exposure to elevated [O_3_]; both have been used as indicators of stress perception (Castagna and Ranieri, 2009). Greater foliar phenolic content was measured in pea and soybean, but not in common bean (Fig. 4B). Common bean also had the greatest O_3_-mediated decrease in photosynthetic capacity (Figs 1 and 3), which is consistent with the notion that phenolic metabolites may directly scavenge ROS (Rice-Evans *et al.*, 1997; Grace and Logan, 2000). Evidence from garden pea, which had an increase in the abundance of phenolic metabolites (Fig. 4B) and no change in photosynthesis (Figs 1 and 3), also supports this idea. However, photosynthesis was negatively affected by elevated [O_3_] in soybean (Figs 1 and 3) despite a doubling of phenolic content (Fig. 4B). Previous work with another soybean variety did not report any differences in phenolic content with exposure to either chronic or acute O_3_ stress (Gillespie *et al.*, 2011). Such inconsistency between phenolic content and responsiveness of other phenotypes has been previously observed in other species (Catagna and Ranieri, 2009) and questions the reliability of using phenolics-related transcripts and metabolites as biomarkers to predict O_3_ sensitivity. The results presented here do show, however, that transcript abundance changes of phenolics-related biosynthesis genes (Fig. 4A) can be used as general indicators of elevated [O_3_] perception.

As the first line of defence, extracellular antioxidants including ascorbate are thought to contribute to detoxification of O_3_ (Chameides, 1989; Luwe *et al.*, 1993; Burkey *et al.*, 2003; Conklin and Barth, 2004; Cheng *et al.*, 2007). Garden pea maintained the size and redox status of the apoplastic ascorbate pool when exposed to elevated [O_3_], in contrast to common bean, which showed a significant reduction in both the size and redox state of the apoplastic ascorbate pool at elevated [O_3_] (Table 2). Reduced ascorbate in the apoplast needs to be regenerated from oxidized ascorbate (dehydroascorbate; DHA), which is typically performed by glutathione-dependent dehydroascorbate reductase (DHAR) after transport of DHA across the plasma membrane into the cytoplasm (Conklin and Barth, 2004; Foyer and Noctor, 2005). While no change in transcript abundance was seen for *DHAR* homologues (Supplementary Table S3), the pool size of glutathione was greatest in garden pea (Table 2). Significant variation in glutathione pools among dicot species has been previously reported, and in healthy leaves most of the glutathione pool is localized to the mitochondria (Zechmann and Müller, 2010). There, glutathione is important for the maintenance of the redox status to avoid or repair oxidative damage (Marí *et al.*, 2009). Pea had greater rates of respiration in both ambient and elevated [O_3_] than soybean or common bean (Fig. 5), which may in part explain the greater foliar glutathione content. Increased transcript abundance of a cytosolic GR was observed in response to elevated [O_3_] (Table 1), which along with glutathione availability is important for the recycling of reduced ascorbate (Noctor and Foyer, 1998) and would support DHAR activity. Previous studies have demonstrated that GR content is positively correlated with protection from O_3_-induced damage (Tanaka *et al.*, 1990; Chernikova *et al.*, 2000). Other O_3_-reponsive genes also increased transcript abundance specifically in garden pea that would enable GR function and help regulate adequate levels of reduced glutathione, such as *G6PDH* and *GGT* (Ohkama-Ohtsu *et al.*, 2007; Wang *et al.*, 2008; Yang *et al.*, 2014). Collectively, this evidence suggests that garden pea both harnessed the scavenging potential of the apoplastic ascorbate pool in response to elevated [O_3_], and regenerated the reduced ascorbate needed for efficient detoxification or signalling. The fact that no increase in abundance of chloroplastic *GPX6* and *SOD2* transcripts was seen in garden pea further supports this inference. The response of *GPX6* and *SOD2* transcripts to elevated [O_3_] in soybean and common bean (Table 1) indicates that penetration of ROS into chloroplasts was likely, resulting in damage to photosynthetic proteins and decreased photosynthetic rates (Figs 1 and 3).

In response to elevated [O_3_], increased rates of respiration have been observed in soybean (Gillespie *et al.*, 2012), bean (Todd, 1958; Amthor, 1988), and other plant species (Volin and Reich, 1996; Kellomaki and Wang, 1998; Biswas *et al.*, 2008). Increased respiration is hypothesized to supply the energy demands associated with antioxidant scavenging and cellular repair mechanisms (Amthor, 1988). All three of the legume species investigated in this study showed increased mitochondrial respiration in the light (*R*_d_), estimated from *A*/*C*_i_ curve analysis (Fig. 5B). All three species also decreased TNC content at elevated [O_3_] (Table 3), a result in soybean and common bean that may be driven both by decreased photosynthesis and increased respiration. However, in garden pea, photosynthetic capacity was similar in ambient and elevated [O_3_], while respiration rates increased and TNCs decreased, which may be interpreted as further evidence for the hypothesis that increased respiration drives increased antioxidant and defence metabolism. Enhanced expression of transcripts involved in glycolysis, the TCA cycle, and mitochondrial electron transport was observed in all three species (Fig. 5A). In elevated [O_3_], garden pea increased the transcript abundance of NADH dehydrogenase, which is a component of complex I in the mitochondrial electron transport system (Fig. 5A; Supplementary Table S6) and plays an important role in regulating ATP synthesis (Rasmusson *et al.*, 1998). Considering that mitochondria are responsible for maintaining redox homeostasis in response to elevated [O_3_] (Dutilleul *et al.*, 2003), the increased abundance of NADH dehydrogenase transcripts may represent a control point in the transcriptional crosstalk involved with regulating the energy demands of enhanced ROS detoxification. Such a response would facilitate the suite of changes observed in antioxidant metabolism that were described previously for garden pea. However, the change in respiration at elevated [O_3_] may not be solely attributed to greater demand for energy for antioxidant metabolism. All three species also showed decreased SLW in elevated [O_3_] (Table 3). Across a very broad range of functional types and species, SLW is negatively correlated with dark respiration rates (Reich *et al*., 1988). It is possible that the effect of elevated [O_3_] on *R*_d_ is driven by the decrease in SLW at elevated [O_3_]. Amthor (1988) suggested that there are mechanisms which would both enhance and decrease respiration rates in plants exposed to elevated [O_3_], and at the transcriptional level there is evidence in the three legume species studied here that genes associated with respiration both increase and decrease (Fig. 5).

While several mechanisms of O_3_ response were identified that were different among the three legume species, there were also common responses. For example, all three species increased *ALDH* transcript abundance at elevated [O_3_] (Supplementary Table S6). Aldehydes are potentially toxic intermediates generated by several metabolic pathways and can accumulate in response to abiotic stress (Kirch *et al.*, 2004). In *Arabidopsis*, overexpression of *ALDH* improved oxidative stress tolerance owing to its role in reactive aldehyde detoxification, an oxidative process generating NADPH that can be then used in respiratory ATP synthesis (Kotchoni *et al.*, 2006). The fact that all three species increased *ALDH* transcript abundance suggests that aldehyde detoxification may be a universal response to counter the negative effects of growth in elevated [O_3_].

In conclusion, a broad range of O_3_-mediated growth, injury, and physiological responses were observed among the three legume species, from the typical O_3_-mediated reductions in leaf-level photosynthesis and leaf longevity in soybean and common bean to no change in garden pea. Comparing global transcriptomic changes with leaf antioxidant content and redox state identified the induction of transcripts encoding phenolic compounds and a greater quantity of phenolic compounds in pea, as well a reduced proportion of apoplastic ascorbate. Pea also had greater foliar glutathione content than soybean and common bean, greater respiration rates, and enhanced NADH dehydrogenase transcript abundance. These general responses of garden pea could be used to develop screens for more tolerant varieties of soybean and common bean, or used in biotechnology applications to improve their response to elevated [O_3_].

## Supplementary data

Supplementary material are available at *JXB* online.

Supplementary Fig. S1 Quality assurance of RNA used for library preparation.

Supplementary Fig. S2 Volcano plot of *P*-values against the expression ratio between elevated and ambient [O_3_].

Supplementary Fig. S3 The distribution of differentially expressed genes in each of the major functional categories and protein processes sub-categories.

Supplementary Table S1 RNA-Seq summary statistics.

Supplementary Table S2 Transcripts with no detectable reads in ambient [O_3_] that were induced by elevated [O_3_].

Supplementary Table S3 Transcript abundance changes of all ascorbate–glutathione cycle genes.

Supplementary Table S4 Transcript abundance changes of glutathione biosynthesis and catabolism genes.

Supplementary Table S5 Global abundance of glutathionylated proteins.

Supplementary Table S6 Transcript abundance changes of respiration-related genes presented in Fig. 5.

Supplementary Data
