# Supplementary material for: A comparative analysis of transcriptomic, biochemical, and physiological responses to elevated ozone identifies species-specific mechanisms of resilience in legume crops
Source: J Exp Bot. 2015 Aug 31;66(22):7101–12. doi: 10.1093/jxb/erv404 (PMC4765784; doi:10.1093/jxb/erv404)
Supplement: Supplementary Data [file supp_66_22_7101__index.html]

A comparative analysis of transcriptomic, biochemical, and physiological responses to elevated ozone identifies species-specific mechanisms of resilience in legume crops — Supplementary Data 

# A comparative analysis of transcriptomic, biochemical, and physiological responses to elevated ozone identifies species-specific mechanisms of resilience in legume crops

## Supplementary Data

Data files

- Supplementary Data - Supplementary Data
