# Supplementary material for: A comparative analysis of transcriptomic, biochemical, and physiological responses to elevated ozone identifies species-specific mechanisms of resilience in legume crops
Source: J Exp Bot. 2015 Aug 31;66(22):7101–12. doi: 10.1093/jxb/erv404 (PMC4765784; doi:10.1093/jxb/erv404)
Supplement: Supplementary Data [file supp_erv404_jexbot150482_file001.pdf]

Supporting data

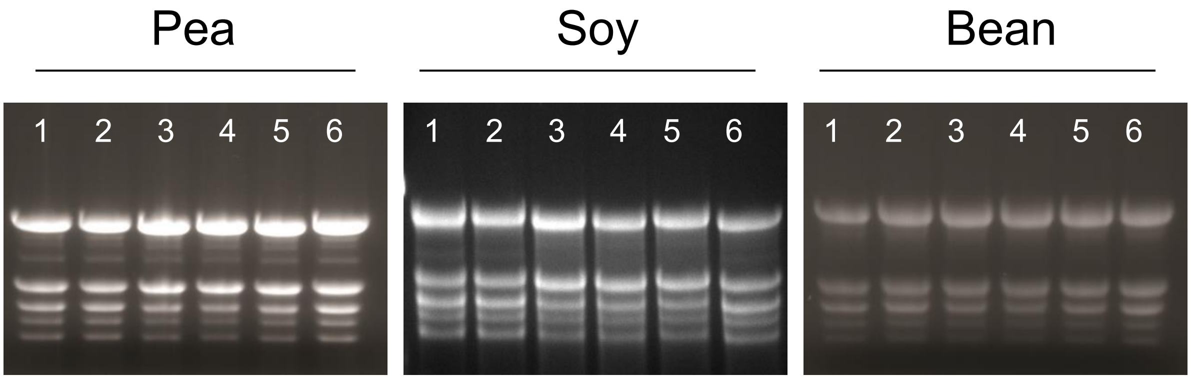

Figure S1 Quality assurance of RNA used for library preparation. Three micrograms of each sample was treated with formaledhyde and run on a 1% agarose gel for 1.5 h at 80 V.

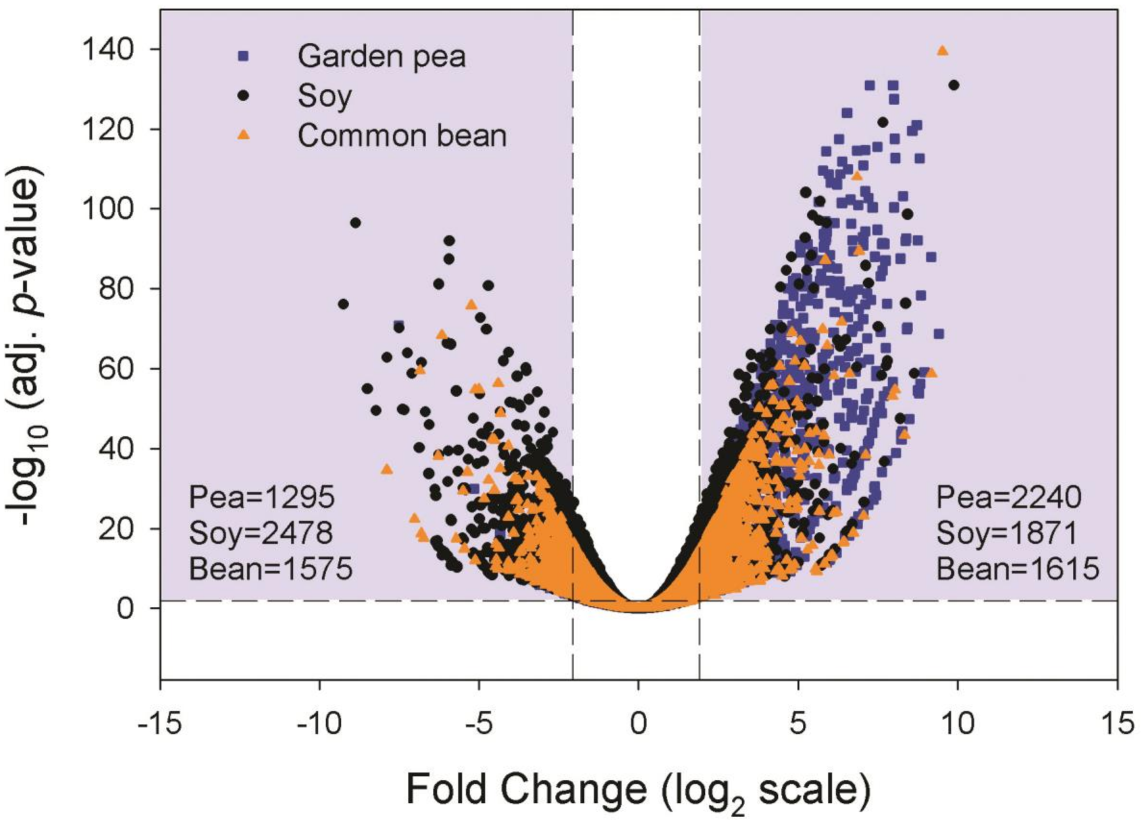

Figure S2 Volcano plot of *p*-values against the expression ratio between elevated and ambient [O_3]. The number of differentially expressed genes, increased and decreased ($\log_2$ FC > 2), are indicated for each species. The horizontal dashed line represents the cutoff ($-\log_{10}(0.05)=1.3$) for significant changes in transcript abundance.

(a)

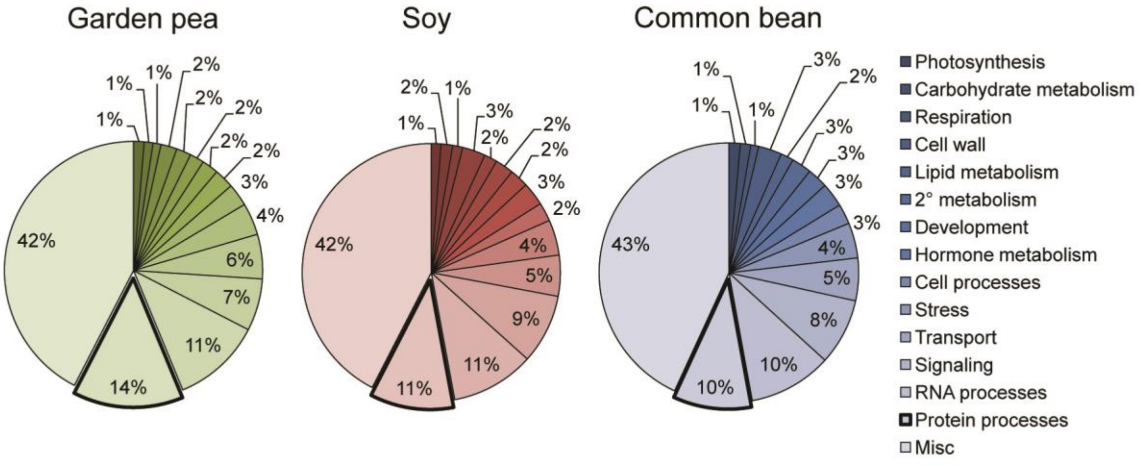

(b)

	Pea		Soy		Bean	
	Up	Down	Up	Down	Up	Down
Protein synthesis	7.1%	5.5%	2.3%	4.7%	1.1%	4.9%
Post-translational modification	26.7%	14.2%	13.2%	16.3%	17.8%	10.4%
Protein degradation	19.6%	10.4%	9.8%	9.4%	9.5%	7.3%

Figure S3 The distribution of differentially expressed genes in each of the (a) major functional categories and (b) protein processes sub-categories. The total number of genes per functional category varied in each species. Therefore, the percent of genes that were differentially expressed was calculated to allow for inter-specific comparisons.

Table S1 RNA-Seq summary statistics. The quality score (Q.S.) was obtained from the Illumina sequencing error report and represents the probability of a base calling error (a Q.S. of 30 predicts one error in 1,000). Non-duplicate reads map to a single transcriptomic location and only appear once in the sequenced library.

Garden pea

Sample	Treatment	Q.S. (median)	Q.S. (interquartile range)	Total reads	Non-duplicate reads
1	Ambient	34	32-35	58,245,652	8,001,483
2	Elevated	34	31-35	36,079,673	6,704,861
3	Ambient	34	32-35	42,911,559	6,213,225
4	Elevated	34	32-35	36,353,315	6,218,210
5	Ambient	34	31-35	36,007,587	6,823,764
6	Elevated	34	31-35	37,448,377	7,351,586

Soybean

Sample	Treatment	Q.S. (median)	Q.S. (interquartile range)	Total reads	Non-duplicate reads
1	Ambient	34	26-36	31,889,531	5,165,761
2	Elevated	34	26-36	17,575,844	3,622,834
3	Ambient	34	29-37	16,862,414	3,368,993
4	Elevated	34	29-37	28,554,551	4,612,331
5	Ambient	34	28-37	36,408,402	5,337,079
6	Elevated	34	28-37	37,605,167	5,508,196

Common bean

Sample	Treatment	Q.S. (median)	Q.S. (interquartile range)	Total reads	Non-duplicate reads
1	Ambient	34	31-35	38,316,012	8,688,802
2	Elevated	34	31-35	47,585,991	10,584,597
3	Ambient	34	31-35	47,976,364	10,633,370
4	Elevated	34	31-35	41,878,954	10,672,292
5	Ambient	34	31-35	34,733,523	7,874,018
6	Elevated	34	31-35	59,387,779	13,253,090

Table S2 Transcripts with no detectable reads in ambient [O_3] that were induced by elevated [O_3]. Transcript abundance values are presented as reads per kilobase of transcript per million mapped reads (RPKM).

	Functional category	RPKM Ambient	RPKM Elevated	Transcript id
Garden pea	Peroxidase	0	4.31	ID_Pisum_sativum_v2_Contig7212
	Peroxidase	0	2.78	ID_Pisum_sativum_v2_Contig4150
	Peroxidase	0	3.57	ID266398_p.sativum_wa1_contig30731
	Receptor kinase	0	5.40	ID272355_p.sativum_wa1_contig18096
	Regulation of transcription (MYB)	0	12.33	ID292722_p.sativum_wa1_contig16669
	Regulation of transcription (WRKY)	0	9.86	ID275406_p.sativum_wa1_contig26221
	Regulation of transcription (C2H2)	0	7.51	ID283674_p.sativum_wa1_contig32903
	Regulation of transcription (bHLH)	0	7.01	ID281873_p.sativum_wa1_contig30326
	Glycosyltransferase family	0	4.79	ID262818_p.sativum_wa1_contig14496
	Beta glucosidase	0	3.38	ID_Pisum_sativum_v2_Contig5348
	Glycosyl hydrolase family	0	8.94	ID_Pisum_sativum_v2_Contig6473
	Oxidoreductase	0	3.07	ID270141_p.sativum_wa1_contig19537
	Oxidoreductase	0	10.32	ID282545_p.sativum_wa1_contig35694
	Phenylpropanoid metabolism	0	11.72	ID278645_p.sativum_wa1_contig10749
	Phenylpropanoid metabolism	0	1.58	ID278068_p.sativum_wa1_contig10748
	Phenylpropanoid metabolism	0	14.07	ID277864_p.sativum_wa1_contig34316
	Ethylene metabolism	0	11.38	ID278339_p.sativum_wa1_contig27669
	Ethylene signal transduction	0	6.38	ID284862_p.sativum_wa1_contig34217
	Glutaredoxin	0	4.21	ID295196_p.sativum_wa1_contig33563
	Cytochrome P450	0	6.72	ID_Pisum_sativum_v2_Contig5309
	Biotic stress	0	5.94	ID294878_p.sativum_wa1_contig30271
	Potassium transport	0	2.50	ID288752_p.sativum_wa1_contig19514
	Amino acid transport	0	7.80	ID300050_p.sativum_wa1_contig22450
	Phosphate transport	0	27.65	ID270856_p.sativum_wa1_contig33632
	Nudix hydrolase	0	39.09	ID263530_p.sativum_wa1_contig33031
	Nudix hydrolase	0	21.11	ID263472_p.sativum_wa1_contig25469
Soy	Peroxidase	0	7.06	Glyma03g04750.1
	Receptor kinase	0	4.47	Glyma08g21321.2
	Receptor kinase	0	4.55	Glyma09g16930.2
	Receptor kinase	0	1.24	Glyma12g17690.2
	Protein degradation	0	2.32	Glyma10g23510.2
	Lipid metabolism	0	4.69	Glyma12g25950.1
Common bean	Peroxidase	0	3.81	Phvul.003G294000.1
	Peroxidase	0	1.43	Phvul.006G130000.1
	Receptor kinase	0	2.01	Phvul.009G239600.1
	Receptor kinase	0	0.83	Phvul.005G167700.1
	Beta glucosidase	0	1.90	Phvul.005G039100.1
	Oxidoreductase	0	2.55	Phvul.007G113300.1
	Short chain dehydrogenase	0	1.62	Phvul.011G097100.1
	Ethylene metabolism	0	1.29	Phvul.010G103900.1

Table S3 Transcript abundance changes of all ascorbate-glutathione cycle genes. Abundance values are presented as reads per kilobase of transcript per million mapped reads (RPKM). Transcripts in bold font are significantly different in response to elevated [O_3] (false discovery rate adjusted $p \leq 0.05$). Transcript sequences of pea and common bean (Bean) were compared to soybean (Soy) using BLAST, and the most closely related transcripts based on primary sequence are grouped together.

Gene	Species	Transcript id	log_2FC	Ambient RPKM	Elevated RPKM
ASCORBATE OXIDASE [cell wall]	**Soy**	**Glyma05g33470.1**	**2.93**	**3.37**	**25.93**
	**Pea**	**ID296830_p.sativum_wa1_contig18841**	**5.45**	**1.99**	**84.39**
ASCORBATE OXIDASE [cell wall]	Soy	Glyma13g03650.1	-0.31 (n.s.)	18.32	15.14
ASCORBATE OXIDASE [cell wall]	**Soy**	**Glyma20g12150.1**	**-0.99**	**21.01**	**10.70**
	**Bean**	**Phvul.006G011600.1**	**1.11**	**22.53**	**46.41**
	**Bean**	**Phvul.006G011700.1**	**-1.92**	**19.68**	**4.94**
	Pea	ID_Pisum_sativum_v2_Contig4699	-0.12 (n.s.)	145.03	129.09
ASCORBATE PEROXIDASE 1 [cytoplasm]	Soy	Glyma11g15680.5	-0.26 (n.s.)	78.73	67.00
	Bean	Phvul.011G071300.1	0.19 (n.s.)	386.48	421.50
	Pea	ID270485_p.sativum_wa1_contig24721	-0.09 (n.s.)	726.26	661.10
ASCORBATE PEROXIDASE 1 [cytoplasm]	Soy	Glyma12g07780.3	0.068 (n.s.)	192.14	205.72
ASCORBATE PEROXIDASE 3 [microsome]	**Soy**	**Glyma11g08320.1**	**-2.02**	**6.41**	**1.56**
	**Bean**	**Phvul.002G107000.1**	**-2.32**	**4.79**	**0.90**
	Pea	ID290629_p.sativum_wa1_contig26708	-0.27 (n.s.)	8.97	7.21
ASCORBATE PEROXIDASE 3 [microsome]	Soy	Glyma11g11460.1	0.10 (n.s.)	93.79	102.49
ASCORBATE PEROXIDASE 3 [microsome]	Soy	Glyma12g03610.1	0.42 (n.s.)	8.33	11.32
	Bean	Phvul.011G035000.1	0.08 (n.s.)	16.76	16.88
	Pea	ID284080_p.sativum_wa1_contig21676	-0.28 (n.s.)	187.40	149.09
ASCORBATE PEROXIDASE 4 [chloroplast]	Soy	Glyma14g35440.2	0.35 (n.s.)	1.50	1.97
ASCORBATE PEROXIDASE 6 [cytoplasm]	Soy	Glyma06g07180.1	-0.27 (n.s.)	4.50	3.84
	**Bean**	**Phvul.009G093000.1**	**-0.88**	**43.84**	**22.66**
ASCORBATE PEROXIDASE [thylakoid]	Soy	Glyma04g42720.2	0.33 (n.s.)	0.84	1.04
	Bean	Phvul.009G126500.1	-0.65 (n.s.)	2.99	1.80
	Bean	Phvul.009G126500.2	0.18 (n.s.)	13.97	15.10
	Pea	ID_Pisum_sativum_v2_Contig5697	0.01 (n.s.)	154.50	150.41
ASCORBATE PEROXIDASE [thylakoid]	Soy	Glyma06g12020.1	-0.13 (n.s.)	29.81	27.82
CATALASE 2	Soy	Glyma04g01920.1	0.21 (n.s.)	14.93	17.60
	**Pea**	**ID_Pisum_sativum_v2_Contig4094**	**-0.63**	**1378.58**	**853.61**

CATALASE 2	Soy	Glyma04g01920.2	0.18 (n.s.)	2.13	2.40
CATALASE 2	Soy	Glyma06g02040.4	-0.24 (n.s.)	2.16	1.96
	Bean	Phvul.009G011100.1	0.07 (n.s.)	571.15	572.24
CATALASE 2	Soy	Glyma14g39810.1	0.33 (n.s.)	74.56	95.98
CATALASE 2	Soy	Glyma17g38140.1	-0.01 (n.s.)	92.79	93.94
	Bean	Phvul.001G001000.1	0.07 (n.s.)	54.47	54.30
DEHYDROASCORBATE REDUCTASE 2	Soy	Glyma10g43730.1	-0.37 (n.s.)	19.37	15.51
DEHYDROASCORBATE REDUCTASE 2	Soy	Glyma20g38440.1	-0.42 (n.s.)	63.15	48.31
	Bean	Phvul.007G009800.1	-0.03 (n.s.)	57.96	54.31
	Pea	ID_Pisum_sativum_v2_Contig1642	0.57 (n.s.)	1.81	2.61
DEHYDROASCORBATE REDUCTASE 3 [chloroplast]	Soy	Glyma11g33700.3	0.01 (n.s.)	23.53	24.15
DEHYDROASCORBATE REDUCTASE 3 [chloroplast]	Soy	Glyma18g04510.1	-0.09 (n.s.)	71.68	69.03
	Bean	Phvul.001G230400.1	0.24 (n.s.)	87.11	98.06
	Pea	ID_Pisum_sativum_v2_Contig8801	-0.42 (n.s.)	3.83	2.77
	Pea	ID262802_p.sativum_wa1_contig12414	0.01 (n.s.)	24.60	23.83
GLUTATHIONE PEROXIDASE 3	Soy	Glyma05g34490.5	0.35 (n.s.)	4.05	5.34
	Bean	Phvul.002G322400.1	0.46 (n.s.)	93.40	122.63
	Pea	ID281337_p.sativum_wa1_contig33760	0.07 (n.s.)	7.52	7.59
GLUTATHIONE PEROXIDASE 3	**Soy**	**Glyma05g34490.6**	**0.87**	**1.64**	**3.20**
GLUTATHIONE PEROXIDASE 3	Soy	Glyma08g05200.4	0.06 (n.s.)	10.48	11.06
	Pea	ID263199_p.sativum_wa1_contig27565	0.28 (n.s.)	2.89	3.45
GLUTATHIONE PEROXIDASE 4	Soy	Glyma03g30800.1	0.29 (n.s.)	36.79	45.70
	Pea	ID_Pisum_sativum_v2_Contig7800	-0.45 (n.s.)	66.45	46.99
GLUTATHIONE PEROXIDASE 6	**Soy**	**Glyma01g42840.1**	**0.84**	**71.66**	**131.13**
GLUTATHIONE PEROXIDASE 6	Soy	Glyma05g37900.1	0.35 (n.s.)	13.37	17.33
	Pea	ID_Pisum_sativum_v2_Contig1327	0.19 (n.s.)	7.15	7.95
GLUTATHIONE PEROXIDASE 6	Soy	Glyma08g01700.1	0.03 (n.s.)	102.18	106.75
	**Bean**	**Phvul.002G288700.1**	**1.34**	**173.55**	**417.89**
GLUTATHIONE PEROXIDASE 6	Soy	Glyma11g02630.1	0.37 (n.s.)	47.88	63.38
GLUTATHIONE PEROXIDASE 7	Soy	Glyma14g11685.1	-0.06 (n.s.)	5.71	5.43
GLUTATHIONE PEROXIDASE 7	Soy	Glyma17g34110.1	0.03 (n.s.)	17.88	18.57
	Pea	ID270618_p.sativum_wa1_contig03738	-0.07 (n.s.)	184.69	170.34
GLUTATHIONE REDUCTASE [cytoplasm]	Soy	Glyma02g08180.1	0.32 (n.s.)	36.95	47.14
	Bean	Phvul.004G083600.1	0.30 (n.s.)	81.55	95.88
GLUTATHIONE REDUCTASE [cytoplasm]	Soy	Glyma16g27210.1	-0.11 (n.s.)	1.08	1.00

	Bean	Phvul.004G083700.1	0.21 (n.s.)	65.64	72.45
	**Pea**	**ID_Pisum_sativum_v2_Contig8768**	**1.23**	**97.60**	**224.23**
GLUTATHIONE REDUCTASE [chloroplast]	Soy	Glyma02g16010.3	0.70 (n.s.)	1.92	3.15
	Pea	ID_Pisum_sativum_v2_Contig4628	-0.20 (n.s.)	153.54	129.60
GLUTATHIONE REDUCTASE [chloroplast]	Soy	Glyma02g16010.4	-0.13 (n.s.)	2.10	2.03
GLUTATHIONE REDUCTASE [chloroplast]	Soy	Glyma10g03740.5	0.23 (n.s.)	3.22	3.86
	Bean	Phvul.007G177200.1	-0.05 (n.s.)	99.64	91.60
MONODEHYDROASCORBATE REDUCTASE 1	Soy	Glyma0169s00210.1	-0.02 (n.s.)	158.97	160.81
	Bean	Phvul.007G230300.1	0.10 (n.s.)	284.07	289.11
MONODEHYDROASCORBATE REDUCTASE 1	Soy	Glyma10g07820.1	-0.25 (n.s.)	139.46	120.09
	Pea	ID_Pisum_sativum_v2_Contig4113	-0.08 (n.s.)	354.43	325.58
MONODEHYDROASCORBATE REDUCTASE 4	Soy	Glyma16g07970.1	-0.32 (n.s.)	54.20	44.53
	Bean	Phvul.008G155600.1	-0.37 (n.s.)	78.86	58.21
	Pea	ID289338_p.sativum_wa1_contig19560	-0.36 (n.s.)	112.92	84.57
MONODEHYDROASCORBATE REDUCTASE 6	**Soy**	**Glyma05g37430.2**	**1.26**	**1.27**	**3.22**
SUPEROXIDE DISMUTASE 1 [Cu,Zn]	Bean	Phvul.006G097000.1	-0.14 (n.s.)	169.55	146.36
SUPEROXIDE DISMUTASE 2 [Cu,Zn]	**Soy**	**Glyma11g19840.2**	**-1.07**	**10.49**	**4.85**
SUPEROXIDE DISMUTASE 2 [Cu,Zn]	**Soy**	**Glyma12g08650.1**	**-1.68**	**33.11**	**10.37**
	**Bean**	**Phvul.011G086300.1**	**-0.60**	**202.81**	**127.54**
SUPEROXIDE DISMUTASE 2 [Cu,Zn]	**Soy**	**Glyma12g30260.1**	**-2.00**	**22.90**	**5.64**
SUPEROXIDE DISMUTASE 3 [Cu,Zn]	Soy	Glyma16g27020.2	-0.29 (n.s.)	19.12	16.10
	Bean	Phvul.004G081000.1	-0.19 (n.s.)	39.31	32.83
	Pea	ID_Pisum_sativum_v2_Contig4052	-0.24 (n.s.)	82.37	67.20
SUPEROXIDE DISMUTASE 2 [Fe; chloroplast]	Soy	Glyma10g33710.1	0.26 (n.s.)	52.80	64.44
	**Bean**	**Phvul.007G135400.1**	**1.16**	**35.61**	**75.98**
SUPEROXIDE DISMUTASE 2 [Fe; chloroplast]	Soy	Glyma10g33710.2	0.47 (n.s.)	7.33	10.16
SUPEROXIDE DISMUTASE 2 [Fe; chloroplast]	**Soy**	**Glyma20g33880.2**	**0.53**	**346.98**	**512.39**
	**Bean**	**Phvul.007G135400.2**	**0.74**	**2.32**	**3.72**
SUPEROXIDE DISMUTASE 3 [Fe; chloroplast]	Soy	Glyma20g12510.1	0.38 (n.s.)	12.30	16.34
	Bean	Phvul.006G010800.1	-0.33 (n.s.)	20.79	15.90
SUPEROXIDE DISMUTASE 1 [Mn]	**Soy**	**Glyma04g39930.1**	**0.52**	**26.54**	**38.72**
	Bean	Phvul.009G141600.1	0.28 (n.s.)	106.03	123.09
	Pea	ID_Pisum_sativum_v2_Contig4187	-0.04 (n.s.)	104.80	99.06
SUPEROXIDE DISMUTASE 1 [Mn]	Soy	Glyma06g14960.1	0.44 (n.s.)	44.52	61.46

Table S4 Transcript abundance changes of glutathione biosysthesis and catabolism genes. Abundance values are presented as reads per kilobase of transcript per million mapped reads (RPKM). Transcripts in bold font are significantly different in response to elevated [O_3] (false discovery rate adjusted $p \leq 0.05$). Transcript sequences of pea and common bean (Bean) were compared to soybean (Soy) using BLAST, and the most closely related transcripts based on primary sequence are grouped together.

	Species	Transcript ID	$\log_2$ Fold Change	Ambient RPKM	Elevated RPKM
γ-GLUTAMYL TRANSPEPTIDASE	Soy	Glyma11g35990.2	1.04 (n.s.)	0.78	1.34
	**Bean**	**Phvul.001G249200.1**	**-1.34**	**13.23**	**5.01**
	**Pea**	**ID291601_p.sativum_wa1_contig26572**	**4.74**	**1.30**	**34.80**
γ-GLUTAMYL TRANSPEPTIDASE	**Soy**	**Glyma18g02450.1**	**-0.90**	**3.51**	**1.88**
	**Pea**	**ID278435_p.sativum_wa1_contig34236**	**5.21**	**0.65**	**23.87**
GLUTAMATE-CYSTEINE LIGASE	Soy	Glyma08g01750.3	0.02 (n.s.)	37.83	39.22
	Bean	Phvul.002G289200.1	0.18 (n.s.)	67.27	72.48
	Pea	ID_Pisum_sativum_v2_Contig4841	-0.03 (n.s.)	96.07	91.74
GLUTATHIONE SYNTHETASE	Soy	Glyma03g40050.1	-0.41 (n.s.)	22.54	17.31
	Bean	Phvul.006G094500.1	-0.06 (n.s.)	55.27	50.65
	Soy	Glyma19g42600.1	0.03 (n.s.)	9.99	10.32
	Pea	ID286494_p.sativum_wa1_contig25210	-0.29 (n.s.)	62.68	57.01
	Pea	ID295758_p.sativum_wa1_contig30872	-0.24 (n.s.)	56.78	46.40

Table S5 Global abundance of glutathionylated proteins. Protein was extracted from approximately 100 mg of leaf tissue and immunoblotted as described in Duncan *et al.* (2006). For each sample, 2.5μg of protein was loaded and separated on a 10% polyacrylamide-0.1% SDS gel and transferred to a polyvinylidene fluoride fluorescence-specific membrane (Millipore, Bedford, MA, USA). Membranes were then probed using an anti-glutathione primary antibody (Millipore, Bedford, MA, USA) using a 1:5,000 dilution. Detection of immune-complexes was achieved by probing the membrane with Alexa Fluor 680 secondary antibodies (Li-Cor Biosciences, Lincoln, NE, USA) diluted 1:10,000 and imaged with the LI-COR Odyssey Imaging System (Lincoln, NE, USA). Densitometry was measured using ImageJ (NIH) by quantifying the optical density at 680 nm of each sample lane.

	Ambient	Elevated
Garden pea	$2.0 \times 10^6 \pm 3.27 \times 10^5$ (a)	$1.7 \times 10^6 \pm 6.85 \times 10^4$ (a)
Soybean	$1.6 \times 10^6 \pm 3.08 \times 10^5$ (a)	$9.7 \times 10^5 \pm 1.63 \times 10^5$ (b)
Common bean	$8.9 \times 10^5 \pm 6.36 \times 10^3$ (b)	$9.7 \times 10^5 \pm 1.69 \times 10^5$ (b)

Reference

Duncan KA, Hardin SC, Huber SC. 2006. The three maize sucrose synthase isoforms differ in distribution, localization, and phosphorylation. *Plant Cell Physiology* **47,** 959-971.

Table S6 Transcript abundance changes of respiration related genes presented in Fig. 5. Abundance values are presented as reads per kilobase of transcript per million mapped reads (RPKM). All transcripts listed are significantly different in response to elevated [O_3] (false discovery rate adjusted $p \leq 0.05$).

Respiratory pathway	Gene	Species	Transcript id	Top soybean match	log_2FC	Ambient RPKM	Elevated RPKM
Glycolysis	*GLUCOSE-6-PHOSPHATE ISOMERASE* [cytoplasm]	Soy	Glyma06g03560.2		0.48	22.98	32.62
Glycolysis	*PHOSPHOFRUCTOKINASE* [cytoplasm]	Soy	Glyma09g01050.1		-1.35	1.22	0.52
		Soy	Glyma15g11890.1		-1.48	2.00	0.69
		Bean	Phvul.006G145300.1	Glyma15g11890.1	-2.03	5.13	1.19
Glycolysis	*GLYCERALDEHYDE 3-PHOSPHATE DEHYDROGENASE* [cytoplasm]	Pea	ID_Pisum_sativum_v2_Contig36	Glyma04g36860.1	-0.57	53.72	34.80
		Pea	ID_Pisum_sativum_v2_Contig6409	Glyma11g37360.1	0.83	195.20	339.40
		Soy	Glyma03g22790.1		-1.07	12.99	6.29
		Soy	Glyma11g37360.1		0.66	31.46	50.85
		Bean	Phvul.001G259000.1	Glyma11g37360.1	0.65	66.79	99.89
Glycolysis	*PHOSPHOGLYCERATE MUTASE* [cytoplasm]	Pea	ID282363_p.sativum_wa1_contig24234	Glyma01g43030.1	-0.76	89.06	50.53
		Pea	ID290074_p.sativum_wa1_contig28839	Glyma05g10140.4	0.93	11.97	22.15
		Pea	ID282982_p.sativum_wa1_contig26055	Glyma20g37486.1	1.61	7.51	22.31
		Soy	Glyma20g37486.1		1.19	24.86	58.38
Glycolysis	*ENOLASE* [cytoplasm]	Pea	ID286398_p.sativum_wa1_contig19099	Glyma18g22780.1	1.53	12.42	34.93
		Soy	Glyma18g22780.1		0.72	6.95	12.01
		Bean	Phvul.004G127500.1	Glyma09g28100.1	0.74	3.76	6.00
Glycolysis	*PYRUVATE KINASE* [cytoplasm]	Bean	Phvul.004G078400.1	Glyma16g26830.1	-1.43	0.98	0.34
Glycolysis	*PHOSPHO-ENOL-PYRUVATE CARBOXYLASE* [cytoplasm]	Pea	ID_Pisum_sativum_v2_Contig4678	Glyma12g33820.1	0.51	109.52	152.14
		Pea	ID297143_p.sativum_wa1_contig21264	Glyma12g35840.2	1.20	57.72	129.36
		Pea	ID14082_GH719589	Glyma12g35840.2	1.70	20.91	66.20
		Pea	ID_Pisum_sativum_v2_Contig7139	Glyma12g35840.2	1.38	42.10	106.52
		Soy	Glyma06g33380.1		-0.73	32.74	20.07

		Soy	Glyma12g33820.1		-0.58	85.11	58.13
		Bean	Phvul.003G024800.1	Glyma01g22840.2	-1.64	4.22	1.29
		Bean	Phvul.005G066400.1	Glyma12g35840.3	1.55	9.89	27.84
Glycolysis	*PHOSPHOGLUCOMUTASE* [plastid]	Soy	Glyma20g02220.1		-0.59	104.19	70.69
		Bean	Phvul.002G096000.1	Glyma20g02220.1	-1.63	113.76	35.36
Glycolysis	*GLUCOSE-6-PHOSPHATE ISOMERASE* [plastid]	Soy	Glyma02g37590.1		-0.47	49.40	36.47
		Bean	Phvul.008G180200.1	Glyma02g37590.1	-0.60	54.40	34.26
Glycolysis	*PHOSPHOFRUCTOKINASE* [plastid]	Pea	ID296901_p.sativum_wa1_contig28437	Glyma08g38450.1	1.47	6.40	17.29
		Pea	ID_Pisum_sativum_v2_Contig6823	Glyma18g21720.1	1.26	9.86	23.11
		Soy	Glyma01g03040.1		1.84	0.54	1.93
		Soy	Glyma08g03570.1		0.87	4.30	8.21
		Soy	Glyma08g21370.1		-0.82	26.20	15.06
		Bean	Phvul.002G306800.1	Glyma05g36050.3	1.41	9.80	24.99
Glycolysis	*PYRUVATE KINASE* [plastid]	Pea	ID265166_p.sativum_wa1_contig23674	Glyma01g40860.2	-0.53	71.57	47.98
		Pea	ID284266_p.sativum_wa1_contig33550	Glyma01g40860.3	-0.52	70.21	46.98
		Pea	ID_Pisum_sativum_v2_Contig2999	Glyma05g09310.2	0.59	37.28	54.82
		Pea	ID277035_p.sativum_wa1_contig18485	Glyma10g37210.1	-0.81	12.15	6.69
		Pea	ID272847_p.sativum_wa1_contig12510	Glyma10g40110.1	-0.80	8.70	4.83
		Pea	ID_Pisum_sativum_v2_Contig4652	Glyma20g35400.2	-0.50	79.79	54.56
		Soy	Glyma09g23150.1		-0.69	33.17	21.11
		Soy	Glyma16g28980.1		-4.43	5.07	0.24
Glycolysis	*PHOSPHOGLYCERATE MUTASE* [localization unclear]	Pea	ID282363_p.sativum_wa1_contig24234	Glyma01g43030.1	-0.76	89.06	50.53
		Pea	ID290074_p.sativum_wa1_contig28839	Glyma05g10140.4	0.93	11.97	22.15
		Pea	ID286427_p.sativum_wa1_contig20903	Glyma06g11070.1	2.14	41.97	180.49
		Pea	ID282982_p.sativum_wa1_contig26055	Glyma20g37486.1	1.61	7.51	22.31
		Soy	Glyma11g19400.1		-0.99	31.91	16.30
		Soy	Glyma12g09100.1		-0.54	74.65	52.35
		Soy	Glyma13g01970.1		-5.87	5.09	0.08
		Soy	Glyma20g37486.1		1.19	24.86	58.38

		Bean	Phvul.011G090600.1	Glyma11g19400.1	-0.68	76.49	45.67
TCA cycle	*PYRUVATE DEHYDROGENASE* [E1]	Soy	Glyma05g27260.1		-0.83	2.72	1.54
		Soy	Glyma14g10550.1		-0.67	34.09	21.90
TCA cycle	*PYRUVATE DEHYDROGENASE* [E2]	Pea	ID278763_p.sativum_wa1_contig17872	Glyma10g35960.1	0.97	37.46	70.48
		Soy	Glyma01g20720.1		-3.93	4.60	0.31
		Soy	Glyma07g37540.1		0.71	6.27	10.47
		Bean	Phvul.003G106700.1	Glyma17g03110.1	0.90	24.42	43.35
TCA cycle	*PYRUVATE DEHYDROGENASE* [E3]	Soy	Glyma07g37050.2		-1.89	1.47	0.41
		Soy	Glyma15g15310.1		-1.00	18.64	9.45
		Soy	Glyma17g03560.1		-0.71	8.09	5.14
TCA cycle	*CITRATE SYNTHASE*	Pea	ID_Pisum_sativum_v2_Contig4865	Glyma08g16770.1	0.73	71.97	116.75
TCA cycle	*ACONITASE*	Pea	ID_Pisum_sativum_v2_Contig4373	Glyma01g36750.1	0.52	90.88	126.78
		Soy	Glyma12g10580.1		0.57	6.09	9.24
		Soy	Glyma13g38480.1		0.72	6.34	10.74
		Soy	Glyma20g34160.1		1.29	1.77	4.56
TCA cycle	*ISOCITRATE DEHYDROGENASE*	Pea	ID_Pisum_sativum_v2_Contig3045	Glyma14g39160.2	1.01	3.64	7.18
		Pea	ID296188_p.sativum_wa1_contig11295	Glyma14g39160.2	0.67	18.12	28.21
		Bean	Phvul.008G239900.1	Glyma14g39160.2	0.58	8.33	11.91
TCA cycle	*2-OXOGLUTARATE DEHYDROGENASE*	Soy	Glyma10g43610.2		2.39	1.41	7.62
TCA cycle	*SUCCINYL-CoA LIGASE*	Pea	ID_Pisum_sativum_v2_Contig5799	Glyma09g29460.1	0.59	79.53	116.77
TCA cycle	*SUCCINATE DEHYDROGENASE*	Bean	Phvul.003G253200.1	Glyma02g06400.1	0.78	56.15	91.95
TCA cycle	*FUMARASE*	Soy	Glyma10g02040.3		1.01	3.01	6.13
TCA cycle	*MALATE DEHYDROGENASE*	Pea	ID_Pisum_sativum_v2_Contig238	Glyma13g16440.1	-0.81	4.84	2.68
TCA cycle	*MALATE OXIDOREDUCTASE*	Pea	ID284036_p.sativum_wa1_contig18557	Glyma04g09110.3	2.01	38.95	154.13
		Pea	ID_Pisum_sativum_v2_Contig4372	Glyma05g35800.2	0.85	80.66	141.09
		Soy	Glyma01g01180.1		0.96	4.85	9.56
		Soy	Glyma08g21530.1		-1.01	5.89	3.01
		Soy	Glyma16g08460.1		0.49	15.13	21.80
		Soy	Glyma18g46340.1		0.84	1.67	3.07

		Bean	Phvul.009G111500.2	Glyma04g09110.3	2.56	0.16	0.90
		Bean	Phvul.010G094700.1	Glyma07g08110.1	0.83	43.82	74.55
		Bean	Phvul.005G166400.1	Glyma15g02230.1	0.87	72.66	126.61
TCA cycle	*ATP-CITRATE LYASE*	Pea	ID_Pisum_sativum_v2_Contig1835	Glyma09g04000.2	0.61	45.57	67.83
		Soy	Glyma01g03530.1		0.57	39.24	59.64
		Soy	Glyma02g04120.1		0.76	3.32	5.94
		Soy	Glyma09g04000.2		-1.19	1.08	0.47
		Soy	Glyma15g15010.1		-1.22	1.91	0.76
		Bean	Phvul.005G043500.1	Glyma08g17010.4	-0.85	29.78	15.81
TCA cycle	Misc. organic acid transformatons	Soy	Glyma08g17450.1		0.85	4.45	8.22
		Bean	Phvul.005G048000.1	Glyma15g41690.2	0.66	53.29	80.23
		Bean	Phvul.005G048300.1	Glyma15g41690.2	0.67	42.24	63.98
TCA cycle	*CARBONIC ANHYDRASE*	Pea	ID298794_p.sativum_wa1_contig09721	Glyma02g03990.2	0.82	6.59	11.35
		Soy	Glyma01g03720.3		-0.82	6.77	3.79
		Soy	Glyma02g37710.1		1.04	1.41	2.98
		Soy	Glyma02g37710.2		1.66	0.61	2.05
		Soy	Glyma06g19400.1		1.63	2.31	6.95
		Soy	Glyma08g39510.2		0.86	10.91	20.63
		Soy	Glyma19g01050.1		-1.21	7.27	3.17
		Soy	Glyma19g01050.10		-0.84	6.97	3.84
		Bean	Phvul.002G071200.1	Glyma02g03990.2	-1.37	12.74	4.70
		Bean	Phvul.008G181400.2	Glyma02g37710.1	2.33	0.41	2.00
		Bean	Phvul.004G013500.1	Glyma05g08590.1	-0.70	8.23	4.83
		Bean	Phvul.009G178900.1	Glyma06g19400.1	1.09	4.22	8.59
		Bean	Phvul.004G013500.3	Glyma19g01050.11	-0.70	48.05	28.16
Electron transport	*NADH-DEHYDROGENASE* [complex I]	Pea	ID_Pisum_sativum_v2_Contig7774	Glyma04g27580.1	2.43	23.06	121.22
		Pea	ID271967_p.sativum_wa1_contig23606	Glyma12g33520.1	-1.32	3.62	1.38
		Pea	ID266822_p.sativum_wa1_contig10263	Glyma13g26630.1	4.31	0.23	4.33
		Soy	Glyma04g27580.1		0.58	14.57	22.32

Electron transport	*NADH-DEHYDROGENASE* [complex I, carbonic anhydrase]	Pea	ID_Pisum_sativum_v2_Contig2147	Glyma05g38160.1	-0.69	167.66	99.88
Electron transport	*NADH-DEHYDROGENASE* [localization unclear]	Pea	ID266675_p.sativum_wa1_contig28876	Glyma06g47560.1	4.10	15.49	259.81
		Pea	ID_Pisum_sativum_v2_Contig3718	Glyma11g35880.1	-0.82	8.62	4.74
		Soy	Glyma03g37250.1		0.54	22.63	33.63
		Soy	Glyma04g14250.1		2.11	14.99	66.56
		Soy	Glyma06g47560.1		1.24	4.32	10.64
		Soy	Glyma06g47560.2		1.98	1.27	5.45
		Soy	Glyma0776s50.2		-0.56	35.03	24.20
		Soy	Glyma08g41251.1		-1.53	5.94	2.21
		Soy	Glyma1000s00350.1		-0.90	17.27	9.25
		Soy	Glyma1246s00210.1		-1.25	3.49	1.48
		Soy	Glyma15g21361.1		-0.91	24.11	13.07
		Soy	Glyma15g21381.1		-1.01	9.53	4.87
		Soy	Glyma17g23830.1		-0.65	12.87	8.29
Electron transport	NADH-DEHYDROGENASE [type II, external]	Pea	ID_Pisum_sativum_v2_Contig4589	Glyma08g06376.1	1.45	53.24	141.15
		Bean	Phvul.006G192400.1	Glyma15g07290.1	2.41	3.61	18.12
Electron transport	NADH-DEHYDROGENASE [type II, internal matrix]	Pea	ID_Pisum_sativum_v2_Contig3315	Glyma14g37400.1	3.31	6.31	61.72
		Pea	ID_Pisum_sativum_v2_Contig8415	Glyma18g06920.1	-0.75	91.46	52.42
		Soy	Glyma02g39280.2		1.21	3.66	8.62
		Soy	Glyma07g31050.1		-0.74	4.22	2.64
		Bean	Phvul.002G223400.1	Glyma07g31050.1	1.57	8.87	25.19
		Bean	Phvul.002G175300.1	Glyma11g27280.1	-2.41	27.98	4.98
		Bean	Phvul.008G260300.1	Glyma14g37400.1	0.70	13.07	20.23
Electron transport	Electron transfer flavoprotein	Soy	Glyma06g42140.1		0.80	16.41	29.05
		Soy	Glyma10g33110.1		0.78	14.93	26.25
		Soy	Glyma12g16291.1		2.04	0.49	2.07
Electron transport	*ALTERNATIVE OXIDASE*	Pea	ID270489_p.sativum_wa1_contig25191	Glyma04g14800.1	1.24	29.88	68.02

		Pea	ID_Pisum_sativum_v2_Contig2718	Glyma08g07690.1	2.52	3.53	19.75
		Pea	ID272230_p.sativum_wa1_contig10126	Glyma08g07690.1	2.70	2.97	19.04
		Soy	Glyma04g14800.1		0.71	37.69	63.25
Electron transport	*CYTOCHROME C*	Pea	ID281263_p.sativum_wa1_contig28984	Glyma01g44120.1	1.50	5.92	16.40
		Pea	ID281290_p.sativum_wa1_contig30839	Glyma09g38230.1	0.66	105.88	163.37
		Pea	ID_Pisum_sativum_v2_Contig7865	Glyma11g12860.1	-0.71	65.61	38.72
		Pea	ID297249_p.sativum_wa1_contig34819	Glyma11g12860.3	-0.69	54.83	32.57
		Pea	ID17056_EX570908	Glyma12g06050.1	0.61	16.64	24.71
		Pea	ID263334_p.sativum_wa1_contig08262	Glyma12g06050.1	1.02	6.34	12.48
		Bean	Phvul.011G058400.1	Glyma12g06050.1	0.76	14.56	23.66
Electron transport	*CYTOCHROME C OXIDASE*	Pea	ID_Pisum_sativum_v2_Contig2148	Glyma15g01360.1	1.03	6.81	13.51
		Soy	Glyma03g23306.1		-1.03	3.15	1.49
		Bean	Phvul.004G070000.1	Glyma03g23306.1	-1.24	5.20	2.10
Electron transport	*ATPase*	Soy	Glyma12g36106.1		-1.02	93.78	47.05
		Soy	Glyma17g23821.1		-0.90	17.34	9.64
	ATPase	Soy	Glyma2269s00200.1		-0.79	9.77	5.63
Other	Fermentation: *LACTATE DEHYDROGENASE*	Pea	ID274179_p.sativum_wa1_contig22645	Glyma17g14950.1	2.33	11.82	58.61
		Soy	Glyma05g04520.2		1.21	15.62	37.04
		Soy	Glyma09g08150.1		0.53	25.75	37.90
		Bean	Phvul.003G219400.1	Glyma05g04520.2	0.59	26.82	38.78
Other	Fermentation: *PYRUVATE DECARBOXYLASE*	Bean	Phvul.008G243200.1	Glyma02g40550.1	0.60	51.66	74.70
		Bean	Phvul.008G095400.1	Glyma07g18570.1	-0.86	16.98	8.95
		Soy	Glyma07g18570.1		-0.79	13.51	7.97
		Soy	Glyma08g18830.1		-7.35	9.93	0.07
		Soy	Glyma13g30490.1		-1.65	1.20	0.37
		Soy	Glyma14g38860.1		0.80	16.60	29.61
		Soy	Glyma18g43460.1		-0.60	72.13	48.49
Other	Fermentation: *ALDEHYDE DEHYDROGENASE*	Pea	ID270381_p.sativum_wa1_contig10911	Glyma02g05760.1	-1.25	4.71	1.88

		Pea	ID266021_p.sativum_wa1_contig20179	Glyma06g12010.1	4.35	9.84	194.33
		Pea	ID_Pisum_sativum_v2_Contig5230	Glyma13g41480.1	-1.19	83.88	35.24
		Pea	ID295295_p.sativum_wa1_contig08348	Glyma18g18910.1	1.00	3.07	6.06
		Soy	Glyma01g03820.1		1.58	23.80	72.95
		Soy	Glyma02g03870.1		1.23	22.75	54.50
		Soy	Glyma02g05760.1		-0.75	31.34	19.04
		Soy	Glyma04g35220.2		1.54	0.76	2.34
		Soy	Glyma07g09630.1		4.13	0.47	9.08
		Soy	Glyma09g08150.1		0.53	25.75	37.90
		Soy	Glyma09g32170.1		-0.70	28.58	18.06
		Soy	Glyma09g32180.1		1.80	7.15	25.54
		Soy	Glyma12g06130.1		-1.49	6.05	2.13
		Soy	Glyma15g03910.1		-0.96	32.57	16.97
		Soy	Glyma16g24420.1		-0.60	17.66	11.85
		Soy	Glyma18g18910.1		1.11	11.53	25.35
		Soy	Glyma19g01390.1		1.01	6.40	13.06
		Bean	Phvul.002G072100.1	Glyma01g03820.1	1.52	43.70	118.31
		Bean	Phvul.002G314000.1	Glyma05g35350.1	0.70	80.61	124.69
		Bean	Phvul.004G162300.1	Glyma07g09630.1	1.88	1.75	6.01
		Bean	Phvul.009G226700.1	Glyma15g19670.1	0.96	32.31	59.73
		Bean	Phvul.003G199100.2	Glyma17g09860.1	0.80	1.49	2.49
Other	Gluconeogenesis / glyoxylate cycle: *CITRATE SYNTHASE*	Soy	Glyma02g45790.1		0.58	15.81	24.41
		Soy	Glyma17g13730.1		1.21	5.27	12.64
		Bean	Phvul.003G209100.1	Glyma17g13740.1	2.68	13.39	80.64
Other	Gluconeogenesis / glyoxylate cycle: *MALATE DEHYDROGENASE*	Pea	ID263664_p.sativum_wa1_contig13195	Glyma07g30430.1	-0.63	275.08	170.77
		Soy	Glyma11g04720.1		0.56	33.47	50.44
		Bean	Phvul.002G007700.1	Glyma01g40580.2	-3.37	4.42	0.39
Other	Gluconeogenesis / glyoxylate cycle: *PEPCK*	Soy	Glyma01g02330.1		-0.55	14.49	10.10
		Soy	Glyma04g09510.1		0.86	15.68	29.01

		Soy	Glyma09g33650.1		0.63	36.48	57.62
		Bean	Phvul.002G139200.1	Glyma09g33650.1	2.19	40.07	173.50
Other	Gluconeogenesis / glyoxylate cycle: *PYRUVATE DIKINASE*	Soy	Glyma07g38390.2		-0.62	94.03	62.58
Other	Gluconeogenesis / glyoxylate cycle: *ISOCITRATE LYASE*	Bean	Phvul.011G105500.1	Glyma12g10780.1	9.52	0.17	117.68
Other	Pentose phosphate pathway, oxidative: *GLUCOSE 6-PHOSPHATE DEHYDROGENASE*	Pea	ID268005_p.sativum_wa1_contig11567	Glyma02g10710.1	-1.10	3.28	1.51
		Pea	ID_Pisum_sativum_v2_Contig3116	Glyma02g10710.1	-0.55	38.49	25.48
		Pea	ID262852_p.sativum_wa1_contig18926	Glyma16g06850.3	1.91	23.28	85.77
Other	Pentose phosphate pathway, oxidative: *6-PHOSPHOGLUCONOLACTONASE*	Bean	Phvul.009G049200.1	Glyma04g40990.1	-0.89	8.15	4.16
Other	Pentose phosphate pathway, oxidative: *6-PHOSPHOGLUCONATE DEHYDROGENASE*	Pea	ID_Pisum_sativum_v2_Contig5003	Glyma08g28230.1	0.68	145.35	228.24
		Bean	Phvul.004G150100.1	Glyma07g11615.3	0.81	27.24	45.36
		Bean	Phvul.006G127500.1	Glyma15g13865.1	0.94	3.99	7.23
		Bean	Phvul.008G023700.2	Glyma18g51260.1	0.73	2.41	3.87
		Bean	Phvul.004G046100.1	Glyma19g05120.2	-1.99	13.36	3.18
Other	Pentose phosphate pathway, non-reductive: *TRANSALDOLASE*	Soy	Glyma18g53700.1		0.74	2.52	4.37
		Bean	Phvul.002G281000.1	Glyma05g33410.2	1.94	0.25	0.97
		Bean	Phvul.008G003600.1	Glyma08g47790.1	0.64	103.80	153.64
Other	Pentose phosphate pathway, non-reductive: *RIBOSE 5-PHOSPHATE ISOMERASE*	Pea	ID_Pisum_sativum_v2_Contig1969	Glyma03g40640.1	-0.73	101.73	58.90
		Pea	ID299182_p.sativum_wa1_contig21283	Glyma20g26110.2	0.50	84.46	115.44
		Soy	Glyma19g43310.1		0.83	5.22	9.60
		Bean	Phvul.006G122300.1	Glyma15g14460.1	-0.69	25.67	15.23
		Bean	Phvul.002G234600.1	Glyma20g26110.2	-0.62	43.54	27.05
Other	Pentose phosphate pathway: electron transfer	Pea	ID292516_p.sativum_wa1_contig29219	Glyma01g44850.1	-0.57	59.26	38.27
